# Ethylene Regulates Energy-Dependent Non-Photochemical Quenching in Arabidopsis through Repression of the Xanthophyll Cycle

**DOI:** 10.1371/journal.pone.0144209

**Published:** 2015-12-02

**Authors:** Zhong Chen, Daniel R. Gallie

**Affiliations:** Department of Biochemistry, University of California, Riverside, California, United States of America; Universidade Federal de Vicosa, BRAZIL

## Abstract

Energy-dependent (qE) non-photochemical quenching (NPQ) thermally dissipates excess absorbed light energy as a protective mechanism to prevent the over reduction of photosystem II and the generation of reactive oxygen species (ROS). The xanthophyll cycle, induced when the level of absorbed light energy exceeds the capacity of photochemistry, contributes to qE. In this work, we show that ethylene regulates the xanthophyll cycle in Arabidopsis. Analysis of *eto1-1*, exhibiting increased ethylene production, and *ctr1-3*, exhibiting constitutive ethylene response, revealed defects in NPQ resulting from impaired de-epoxidation of violaxanthin by violaxanthin de-epoxidase (VDE) encoded by *NPQ1*. Elevated ethylene signaling reduced the level of active VDE through decreased *NPQ1* promoter activity and impaired VDE activation resulting from a lower transthylakoid membrane pH gradient. Increasing the concentration of CO_2_ partially corrected the ethylene-mediated defects in NPQ and photosynthesis, indicating that changes in ethylene signaling affect stromal CO_2_ solubility. Increasing VDE expression in *eto1-1* and *ctr1-3* restored light-activated de-epoxidation and qE, reduced superoxide production and reduced photoinhibition. Restoring VDE activity significantly reversed the small growth phenotype of *eto1-1* and *ctr1-3* without altering ethylene production or ethylene responses. Our results demonstrate that ethylene increases ROS production and photosensitivity in response to high light and the associated reduced plant stature is partially reversed by increasing VDE activity.

## Introduction

Ethylene is involved in regulating multiple aspects of plant development, most notably, fruit ripening, cell expansion, programmed cell death, and organ senescence and is involved in several biotic and abiotic stress responses [1, 2, 3, 4, 5, 6, 7]. Ethylene is produced from methionine by its conversion to *S*-adenosylmethionine (AdoMet) by *S*-adenosylmethionine synthase which 1-aminocyclopropane-1-carboxylate synthase (ACS) converts to methylthioadenosine (MTA) and 1-aminocyclopropane-1-carboxylate (ACC) [8]. ACC oxidase (ACO) then oxidizes ACC to produce ethylene.

Ethylene is perceived following its binding to endoplasmic reticulum-localized receptors [9], of which five different types (i.e., ETR1, ERS1, EIN4, ETR2, and ERS2) are expressed in Arabidopsis [7, 10, 11, 12, 13]. As negative regulators, ethylene receptors, together with the CTR1 Raf-like kinase, signal to repress ethylene responses in the absence of ethylene [14, 15, 16]. Ethylene binding to the N-terminal membrane domain of receptors results in loss of signaling from the receptors and CTR1 which relieves the repression of the downstream components of the ethylene response pathway and induces expression of genes involved in ethylene responses [17, 18, 19, 20].

The role that ethylene plays in regulating photosynthesis has been controversial. Repression of photosynthetic activity is often associated with abiotic stress responses, although for some, such as drought, this repression is thought to be principally a result of abscisic acid (ABA)-mediated stomatal closure which limits CO_2_ diffusion into the leaf interior [21, 22, 23]. Early reports suggested that exogenous ethylene inhibited photosynthesis by promoting stomatal closure [24, 25, 26, 27, 28] but ethylene has also been shown to mediate auxin-induced stomatal opening [29, 30] or to have no effect [31, 32]. Genetic studies have suggested that ethylene delays ABA-induced stomatal closure in Arabidopsis by inhibiting the ABA signaling pathway [33] while promoting stomatal closure when present alone [34, 35].

Recent work has suggested that ethylene stimulates photosynthesis independently of stomatal effects [36, 37]. Although reduced rates of CO_2_ assimilation were observed in leaves of ethylene insensitive plants, the differences were small and observed during hydroponic growth which is unlikely to mimic normal growth conditions. The lack of consensus of whether ethylene regulates aspects of photosynthetic functioning may be due to differences in the species employed, differences in growth conditions used, differences in stomatal effects, as well as the use of plants treated with exogenous ethylene versus ethylene insensitive transgenics expressing heterologous mutant ethylene receptors. Studies of the regulation of photosynthesis by ethylene can also be confounded by the use of ethylene mutants when differences in cell density resulting from differences in cell size are not taken into consideration.

The activity of the transcription factors ETHYLENE INSENSITIVE3 (EIN3) and EIN3- enhances Arabidopsis seedling greening during the transition from skotomorphogenesis to photomorphogenesis specifically by inducing expression of the chlorophyll biosynthetic enzymes PROTOCHLOROPHYLLIDE OXIDOREDUCTASE A and B (PORA/B)[38, 39]. EIN3/EIL1 also cooperate with PHYTOCHROME-INTERACTING FACTOR1 (PIF1) to prevent photo-oxidative damage during seedling de-etiolation. However, EIN3 also promotes leaf senescence in adult leaves [40], suggesting that ethylene has specific roles regulating leaf function depending on the developmental stage of a leaf.

Maize mutants defective in ACC synthase (ACS) expression, which experience lower ethylene production without altered leaf size, exhibit increased chlorophyll content in young as well as old leaves, delayed leaf senescence, and increased CO_2_ assimilation without a significant increase in stomatal conductance [41]. These observations suggest that ethylene negatively regulates photosynthetic activity under normal growth conditions independent of stomatal behavior. Even more significant differences in the rates of CO_2_ assimilation were observed in ACS deficient maize during conditions of water stress, although under these conditions, greater stomatal conductance in the mutants may have accounted for some of the differences in CO_2_ assimilation [41]. These results suggest that ethylene functions to inhibit photosynthesis, either directly or indirectly.

Photosynthesis converts absorbed light energy into chemical energy. The capacity of a plant to use absorbed light energy for photochemistry, however, is limited. Consequently, plants have evolved mechanisms to avoid the over reduction of the photosystems that would otherwise result in the generation of triplet state chlorophyll (^3^Chl*) that can transfer energy to ground-state O_2_ to produce highly destructive singlet oxygen (^1^O_2_*) and other reactive oxygen species (ROS) [42, 43]. Over reduction of the photosystems can also result in the generation of destructive ROS through the Mehler reaction [44]. One mechanism that functions to prevent the over excitation of photosystem (PS) II involves pH-dependent, feedback de-excitation of singlet excited chlorophyll molecules in PSII. This feedback de-excitation, referred to as qE, is one of several processes collectively referred to as non-photochemical quenching (NPQ) of chlorophyll fluorescence. The magnitude of qE is determined by the size of the transmembrane proton gradient (ΔpH) which is generated by coupled photosynthetic electron transport [45, 46]. In addition to qE, quenching associated with state transition (qT), photoinhibition (qI), and the recently described qZ, which is distinct from qT, also contributes to NPQ [47]. Under moderate light conditions, qE predominates to dissipate excess excitation energy absorbed in the PSII antenna pigment bed as heat [48]. Thus, the qE component of NPQ plays a key role in regulating light harvesting and photosynthetic performance to protect against PSII photoinhibition. qE requires de-epoxidized xanthophyll pigments [49], PsbS which is a PSII subunit belonging to the light harvesting complex protein superfamily [50], and an acidic thylakoid lumen [51, 52]. While the qE component of NPQ predominates during normal growth, stress conditions can inhibit photosynthesis and limit the amount of absorbed light energy that can be used for photochemistry. Such conditions can result in an elevated induction of qE in an attempt to dissipate excess excitation energy to protect against the over reduction of PSII. Under conditions of excess light, however, qI, which includes quenching resulting from damage to PSII reaction centers [53], can also contribute substantially to NPQ and is either irreversible or slowly reversible [54]. Abiotic stresses, such as drought, high or low temperatures, or exposure to salt can reduce CO_2_ diffusion into the leaf interior which increases ROS generation and photodamage [55].

The xanthophyll cycle contributes significantly to qE and involves the light-induced de-epoxidation of violaxanthin (V) to antheraxanthin (A) and zeaxanthin (Z) by violaxanthin de-epoxidase (VDE). The generation of Z is critical for the full induction of NPQ and is involved in singlet oxygen scavenging as well as chlorophyll quenching [49, 56, 57, 58, 59]. Lutein has also been implicated in the induction of NPQ as mutants with reduced lutein content exhibit diminished NPQ [60, 61, 62, 63, 64, 65, 66]. Lutein has been suggested to contribute to the structural change of the light harvesting complex II (LHCII) to a trimeric form needed for NPQ, which is dependent on the light-induced transthylakoid membrane pH gradient [64, 67]. A direct role of lutein has also been suggested by the observation that an elevated level of lutein can substitute for Z in qE by quenching singlet-excited chlorophyll through the formation of radical cations [65]. Neoxanthin also contributes to tolerance to high light [67, 68].

VDE, a 43 kD protein encoded by the nuclear gene, *NPQ1*, is transported to the thylakoid lumen where its activity is light regulated. While inactive in the dark, VDE is activated by the reduction in pH resulting from proton pumping across the thylakoid membrane that occurs commensurate with the light-driven electron movement through the photosynthetic electron transport chain. As a consequence, VDE associates with the thylakoid membrane where it interacts with its substrate V [69, 70, 71]. The activation of VDE in the acidified lumen also involves a conformational change of the protein and its dimerization enables simultaneous access to the two epoxide rings of V for their de-epoxidation [72].

The xanthophyll cycle not only protects PSII function but also protects photosynthetic membranes against photooxidation [73]. The absence of de-epoxidized xanthophyll pigments such as Z and A increased the sensitivity of thylakoid membranes lipids to ROS such as ^1^O_2_ [73]. The function of de-epoxidized xanthophyll pigments in protecting against ROS-induced membrane damage was underscored by the observation that the VDE-deficient mutant, *npq1*, which lacks de-epoxidized xanthophyll pigments, was more photosensitive than the PsbS-deficient mutant, *npq4*, which lacks NPQ but has normal levels of de-epoxidized xanthophyll pigments [73]. Moreover, loss of PSII function is correlated with photodamage to thylakoid membranes [74, 75], suggesting that de-epoxidized xanthophyll pigments may protect PSII by at least two means: quenching singlet-excited chlorophyll (qE) and by protecting against photodamage to thylakoid membranes. De-epoxidized xanthophyll pigments such as Z may function by scavenging triplet chlorophyll (^3^Chl), ROS, or free radicals directly to prevent membrane damage [76, 77, 78, 79].

In order to investigate how ethylene might affect photosynthetic processes, we employed mutants altered in ethylene production or signaling in Arabidopsis. *ethylene overproducer 1–1* (*eto1-1*) produces elevated levels of ethylene [14, 80, 81]. ETO1 interacts with type 2 ACC synthases and reduces their activity either through direct inhibition or through promoting their degradation in a proteasome-dependent manner [82]. Loss of ETO1 results in constitutively active type 2 ACS and increased ethylene evolution. When grown in light, *eto1-1* plants are significantly smaller than wild-type plants [80]. The loss of CTR1 expression in the *constitutive triple response 1–3* (*ctr1-3*) mutant disrupts the ability of ethylene receptors to repress the activity of the downstream components of the ethylene response pathway, resulting in a constitutive ethylene response [14]. The *ctr1-3* mutant is characterized by a substantial reduction in cell size and plant stature, greater than that exhibited by *eto1-1* plants [14]. In contrast, the *ethylene insensitive 2–5* (*ein2-5*) mutant is insensitive to ethylene due to the lack of EIN2 expression which is epistatic to CTR1 [19] and is required to activate ethylene responses [83].

In this study, we demonstrate that increased ethylene signaling affects the xanthophyll cycle directly and indirectly. *eto1-1* and *ctr1-3* exhibit aberrant induction of NPQ following exposure to light which can be corrected by inhibiting ethylene perception, e.g., in *eto1-1*. The defects in qE and qI observed in these mutants were associated with an impaired functioning of the xanthophyll cycle resulting from reduced expression and activation of VDE, a reduction in PsbS expression, and a lower transthylakoid membrane pH gradient, and an increase in superoxide production. *eto1-1* did not exhibit the compensating increase in α-tocopherol and ascorbic acid content that occurs in *npq1* which is a VDE null mutant. Restoring VDE activity in *eto1-1* and *ctr1-3* reduced superoxide production and photosensitivity upon exposure to high light. Restoring VDE activity also reversed the small stature of *eto1-1* and *ctr1-3* specifically under high light growth conditions without affecting ethylene production or responsiveness. Restoring VDE activity in *eto1-1* and *ctr1-3* did not improve growth under low light, implicating ROS as contributing to the small stature of *eto1-1* and *ctr1-3* under high light conditions. These results demonstrate that ethylene represses functioning of the xanthophyll cycle while increasing ROS production and reversing these effects improves growth under high light.

## Materials and Methods

### Plant material and transformation

Col-0 Arabidopsis was used throughout this study. Seed of Col-0, *eto1-1*, and *ctr1-3* were obtained from Dr. Paul Larsen. After surface-sterilization and cold treatment at 4°C for 4 days in the dark, seeds were planted on 0.25 x MS agar plates with or without ACC at the concentrations indicated and grown at 20°C in a plant growth room supplemented with Sylvania Gro-Lite fluorescent bulbs (Sylvania, Danvers MA, USA) at a photon flux density (PFD) of 100 μmol photons m^-2^ s^-1^. For adult plants, seeds were germinated on medium for 1 week and transferred to soil and grown under a 16 h light cycle at 21°C in a plant growth chamber at 250 PFD. Wild-type Arabidopsis was transformed at bolting using *Agrobacterium* and the binary vector, pBI121. The primary inflorescence was removed and secondary inflorescences were allowed to initiate before infiltration. Inverted plants were dipped into the infiltration medium containing the Aglo1 strain of *Agrobacterium* containing the transgene. Infiltrated plants were kept on their side for one day and allowed to continue to flower in an upright position in the same growth room. Seeds of infiltrated plants were collected and screened on 0.25 x MS plates containing 50 μg/ml kanamycin and 500 μg/ml vancomycin.

For high light experiments, leaves that had been dark-adapted for 16 hours were floated on ice-containing water and exposed to high light (1300 PFD as supplied from high output sodium light) or sunlight (1900 PFD) for the times indicated. F_o_ and F_m_ were measured immediately before the high light exposure and at time points during recovery. For the analysis, leaves with similar initial F_v_/F_m_ values were used.

### Imaging NPQ

The induction of NPQ and its relaxation were performed using an IMAGING-PAM M-Series Chlorophyll Fluorometer (Heinz Walz GmbH, Effeltrich, Germany). Fluorescence measurements of plants dark-adapted overnight were taken using a relative humidity of 50% and an ambient level of CO_2_. At the start of each experiment, the leaf was exposed to 2 min of far-red illumination (1 PFD) for the determination of F_o_ (minimum fluorescence in the dark-adapted state). False color was applied to the images in a range from black (representing a value of 0) to purple (representing a value of 1). For inhibition of PSII quantum yield measurements, F_v_/F_m_ was measured immediately after the actinic light was turned off and continued for 25 min at 1 min intervals. Inhibition of PSII quantum yield was calculated from (F_v_/F_m_ of reference − F_v_/F_m_ of sample)/(F_v_/F_m_ of reference) where an area selected from a non-stressed WT plant was used as the reference.

### Gas exchange and fluorescence measurements

Gas exchange and fluorescence measurements were performed using a LI-COR Li-6400 portable photosynthesis system (LI-COR, Lincoln, NE) with LI-6400-40 leaf chamber, a relative humidity of 50%, and ambient level of CO_2_. Fluorescence measurements were taken using overnight dark-adapted leaves. At the start of each experiment, the leaf was exposed to 2 min of far-red illumination (1 PFD) for the determination of F_o_ (minimum fluorescence in the dark-adapted state). Saturating pulses (0.8 s of 5000 PFD) were applied to determine the F_m_ or F_m_’ values. Actinic light, consisting of 90% of red light (λ = 630 ± 20 nm) and 10% blue light (λ = 470 ± 20 nm) was provided by LED (light emission diode) sources. F_s_ is the steady fluorescence yield during actinic illumination. F_o_’ (minimum fluorescence in the light-adapted state) was determined in the presence of far-red (λ = 740 nm) light after switching off the actinic light. A total of four to six samples were measured in each experiment. All data presented were calculated from at least three independent measurements. Conventional fluorescence nomenclature was used [84]. NPQ was calculated from (F_m_-F_m_’)/F_m_’, φPSII from (F_m_’-F_s_’)/F_m_’, qP from (F_m_’-F_s_)/(F_m_’-F_o_’), and the electron transport rate (ETR) from φPSII *f *α_leaf_, where *f* is the fraction of absorbed quanta that is used by PSII and is typically assumed to be 0.5 for C3 plants; α_leaf_ is leaf absorbance. NPQ_f_ and NPQ_s_ were determined as described [85].

### Chlorophyll, α-tocopherol, and xanthophyll pigment measurements

Chlorophyll a and b were measured spectrophotometrically as described [86]. Leaf samples were ground in liquid nitrogen and extracted with 90% (v/v) acetone. The absorbance at 664 and 647 nm was determined and used to calculated chlorophyll a and b content by the equations: Chl a = 11.93A_664_-1.93A_647_ and Chl b = 20.36A_647_-5.50A_664_, respectively. Each experiment was repeated 2–3 times and representative results presented.

Xanthophyll pigments were extracted with 100% acetone under dim light and were separated on a Spherisorb ODS-1 column (Alltech) as described [87] using solvent A-1: acetonitrile:methanol:0.1 M Tris-HCl pH 7.5 (72:8:3) and solvent B: methanol:hexane (4:1). Pigments were identified by the retention time of standards using a photodiode-array detector and were quantified using reported extinction coefficients [87].

For α-tocopherol, young Arabidopsis leaves were extracted with 1 ml methanol, dried, and re-dissolved with 0.2 mL ethanol. Samples were filtered (0.2 μm Whatman GD/X) and loaded onto a Spherisorb ODS-1 column (Alltech) which was eluted with a gradient of hexane (solvant A) methyl-t-butyl ether (solvent B) as described [88]. α-Tocopherol content was calculated using a standard curve of α-tocopherol.

### qPCR analysis

Plant material was frozen in liquid nitrogen, ground to a fine powder, and 100 mg was resuspended in 1 ml TRIZOL^®^ Reagent (Invitrogen, Carlsbad CA, USA). Following centrifugation, the supernatant was extracted with 200 μl chloroform and centrifuged to separate the phases. RNA was precipitated from the aqueous phase using isopropyl alcohol, the RNA pellet washed with 75% ethanol and resuspended in RNase-free H_2_O. 1 μg RNA was used to obtain the first-strand cDNA by Omniscript RT Kit (Qiagen, Valencia CA, USA) in a 20 μl reaction. The qPCR analysis was performed using a iQ5 Real-Time PCR Detection System (Bio-Rad, Hercules CA, USA) in 25 μl reactions containing 1x SYBR Green SuperMix 500 nM forward and reverse primers and 10 ng cDNA. ROX was used as the passive reference dye. Reactions were carried out using the following conditions: 95°C/5 min (1 cycle); 95°C/30 sec, 55°C/30 sec, 72°C/30 sec (35 cycles). To detect the presence of *NPQ1*, a forward primer, NPQ1-F1, 5’-ATGACTGGTATATCCTGTCATC-3’, and a reverse primer, NPQ1-R1, 5’-CGTTCTAATGAATGTGCTGAAG-3’ were used. To detect the presence of *NPQ4*, a forward primer, NPQ4-F1, 5’-TATGATCGGTTTCGCTGCATC3’, and a reverse primer, NPQ4-R1, 5’-CAACAGAGTGAACAAGATGAAG-3’ were used. Protein phosphatase PP2A (At1g13320) was used as the reference gene for the quantitation of *NPQ1* and *NPQ4* expression in Arabidopsis leaves. To detect the expression of PP2A, a forward primer, PP2A-FW, 5’-AGTATCGCTTCTCGCTCCAG-3’ and a reverse primer, PP2A-RV, 5’-GTTCTCCACAACCGCTTGGT-3’ were used. The efficiency of PCR was determined by five 10-fold serial dilutions of the template DNAs in triplicate. Three biological replicates were used for each target gene.

### Western analysis

Chloroplasts were prepared as described [89]. Chloroplasts were sonicated and one volume of 2 x SDS-PAGE loading buffer was added. Samples equivalent to 20 μg of total chlorophyll were used for the Western analysis of VDE, 5 μg for the analysis of PsbS, and 20 ng for the analysis of the Rubisco large subunit. Protein extracts were resolved using standard SDS-PAGE and the protein transferred to 0.22 μm PVDF membrane by electroblotting. Following transfer, the membranes were blocked in 5% milk in TPBS (0.1% TWEEN 20, 137 mM NaCl, 2.7 mM KCl, 10 mM Na_2_HPO_4_, 1.4 mM KH_2_PO_4_, pH 7.4) followed by incubation with antiserum raised against VDE, PsbS, or the large subunit of Rubisco diluted 1:1000 in TPBS with 1% milk for 1.5 hrs. The blots were then washed twice with TPBS and incubated with goat anti-rabbit horseradish peroxidase-conjugated antibodies (Southern Biotechnology Associates, Inc., Birmingham, AL) diluted 1:20,000 for 1 hr. The blots were washed twice with TPBS and the signal detected using chemiluminescence (Amersham Corp., Piscataway, NJ).

### Ascorbate measurements

HPLC analysis of ascorbate acid was performed as previously described [90]. Samples were ground with liquid nitrogen and approximately 0.1 g of leaf powder was extracted with 0.5 ml of 0.2% metaphosphoric acid contaning 0.54 mM Na_2_EDTA and 0.01% polyvinylpolypyrrolidone. Extracts were centrifuged at 12,000 x g for 10 min at 4°C to remove debris. The supernatant was filtered (0.25 μm) and 20 μl was loaded onto a C-18 ODS column and eluted with isocratic mobile phase (20 mM NaAc, 0.54 mM Na_2_EDTA, 1.5 mM N-octylamine) at a flow rate of 0.5 ml/min. For dehydroascorbic acid analysis, the extract was neutralized with 0.5 M NaOH to pH 6.5, reduced with 2.5 mM glutathione and 2 ng of recombinant dehydroascorbate reductase for 20 min at room temperature. Ascorbate was detected by a Waters 996 photodiode array detector at 265 nm using L-ascorbate as a standard to generate a standard curve.

### VDE, ZE, and CA activity assays

To measure VDE activity, the thylakoid membrane fraction was isolated from Arabidopsis leaves essentially as described [89]. VDE activity was measured in a reaction containing 10 μL of 1 μm violaxanthin in methanol, 25 μL of 300 μM monogalactosyldiacylglycerol (Lipid Products, South Nutfield, UK) in methanol, 550 μL of 0.2 M sodium citrate pH 5.1, and 20–50 μL of the thylakoid membrane fraction (equivalent to 25–50 μg Chl a). The reaction mixture was vortexed, incubated for 5 min at 30°C, and initiated following the addition of 6 μL of 3M sodium ascorbate. After 4–10 min, the reaction was stopped by the addition of 1N NaOH, centrifuged at 20,000 x g for 2 min, and the pellet containing the lipids and pigments analyzed by HPLC as described [91].

To measure ZE activity, the chloroplast fraction was prepared and ZE activity measured as described [92]. Lysed chloroplast extract equivalent to 18 μg Chl a was added to a reaction containing 0.5 μm zeaxanthin, 400 mM sorbitol, 50 mM HEPES-NaOH pH 7.2, 16 mM sodium ascorbate, 0.5 mM NADPH, and 0.3 mg/ml BSA. The reaction was incubated at room temperature in the dark for 40 min. Total pigments were extracted with methanol, dried, and re-dissolved with acetone. The pigments were determined by HPLC.

To measure carbonic anhydrase (CA) activity, total leaf soluble extract and the chloroplast fraction was prepared and the CA assay performed essentially as described [93]. Extract was added to a reaction containing 100 mM potassium phosphate pH 7.7, and 10 mM DTT. The reaction was initiated following the addition of 3 ml of CO_2_-saturated water. While stirring, the decrease in pH over time was monitored. Units of CA activity were calculated as (*T*b/*T*e)-1, where *T*b and *T*e represent the time (in sec) for the pH to drop from 7.5 to 7.0 in the control and sample reactions, respectively.

Three to four biological replications were assayed for each of these assays. The average and standard derivation was reported.

### Luciferase assay

Cell extract representing the soluble protein fraction prepared from seedlings in luciferase assay buffer [20 mM Tricine, pH 7.8, 1.07 mM (MgCO_3_)_4_Mg(OH)_2_-5H_2_O, 2.67 mM MgSO_4_, 0.1 mM EDTA, 33.3 mM DTT, 270 μM CoA, and 500 μM ATP (Promega)], and the reaction was initiated with the injection of 100 μL of 0.5 mM luciferin in luciferase assay buffer. Photons were counted using a Monolight 2010 Luminometer (Analytical Luminescence Laboratory, San Diego, CA). Each mRNA construct was assayed in duplicate and the average is reported. Protein concentration was determined as described [94].

### Superoxide assay

The rate of superoxide production was measured spectrophotometrically as described [95, 96]. 0.5 cm Arabidopsis leaf discs were infiltrated with 10 ml of 10 mM citrate buffer pH 6.0 containing 50 μM XTT, i.e., sodium 3’-[1-(phenylamino)-carbonyl-3, 4-triazolium]-bis(4-methoxy-6-nitro) benzenesulfonic acid hydrate, and exposed to sunlight (about 1900 PFD) under constant temperature. The rate of superoxide production in the leaf samples was monitored spectrophotometrically every 10 min at 470 nm (extinction coefficient of 2.16 x 10^4^ M^-1^ cm^-1^) for 1 hr.

### Determination of the transthylakoid membrane pH gradient

Intact chloroplasts from WT or mutant Arabidopsis were isolated as described [97]. Chloroplasts equivalent to 15 μg chlorophyll were added to 50 mM Hepes-KOH, pH 7.8, 5 mM MgCl_2_, 330 mM sorbitol, 0.4 μM 9-AA to a final volume of 1 ml. Fluorescence from 9-AA was monitored with a modular spectrofluorimeter (Fluorolog-3, Horiba Scientific, Edison, NJ) using an excitation wavelength of 365 nm and fluorescence of 9-AA detected by scanning from 450 to 460 nm at 0.02 sec intervals. Actinic light was supplied with either high-power LED lights or with a 150W reflector spot lamp. Only the uncharged form fluoresces and moves freely across the thylakoid membrane whereas its protonated form neither fluoresces nor moves across membranes. At physiological pH, the protonated form of 9-AA (K_a_ = 1.26 x 10^-10^) predominates. Following the generation of a transthylakoid membrane pH gradient, the 9-AA accumulates in the thylakoid lumen where it is protonated and quenched. The amount of 9-AA that moves into thylakoid lumen is determined from the quenching of 9-AA fluorescence from which the ΔpH is calculated using the equation: [H^+^]_in_ = K_a_ x [AH^+^]/[A]_in_, where [H^+^]_in_ is the proton concentration of the thylakoid lumen; K_a_ = 1.26x10^-10^; [AH^+^] is the concentration of protonated 9-AA; and [A]_in_ is the concentration of uncharged 9-AA in the thylakoid lumen [98]. The ΔpH is determined from the difference between the pH of the thylakoid lumen and the buffer.

### Determination of the quantum requirement for photosynthesis

The quantum requirement was determined using a LI-COR Li-6400 portable photosynthesis system in which chlorophyll fluorescence and the rate of CO_2_ assimilation in leaves from adult plants was measured simultaneously under a series of actinic light levels (from 0 up to 1200 PFD), under low oxygen air (2% oxygen, 98% nitrogen) supplied with 400 ppm CO_2_. The quantum yield of PSII is calculated by φPSII = (F_m_’-F_s_)/F_m_’, where F_m_’ is the maximal fluorescence during a saturating light flash, and Fs is steady-state fluorescence. The quantum yield of CO_2_ assimilation is calculated by φCO_2_ = (A-A_dark_)/*I*α_leaf_ where A is the assimilation rate, A_dark_ is dark assimilation rate (both with units of μmol CO_2_ •m^-2^ •s^-1^, but A_dark_ is a negative value). *I* is the incident photon flux density (μmol •m^-2^ •s^-1^), and α is leaf absorbance (a value of 0.85 is used). The values of φPSII were then plotted against φCO_2_ values, and the quantum requirement calculated from the slope. Each data point was the average of four to six individual measurements.

### Ethylene determination

Ethylene was measured from whole seedlings which were placed in glass vials and capped with a rubber septum. Following a 2 hour incubation, 0.9 ml of headspace was sampled from each vial and the ethylene content measured using a 6850 series gas chromatography system (Hewlett-Packard, Palo Alto, CA) equipped with a HP Plot alumina-based capillary column (Agilent Technologies, Palo Alto, CA), which can detect as little as 10 nl/l (10 ppb) ethylene. The ethylene peak was identified as that which had the same retention time as pure ethylene. Tissue fresh weight was measured for each sample. Three to four replicates were measured and the average and standard deviation reported.

## Results

### Increased ethylene signaling results in an aberrant induction of non-photochemical quenching

**Fig 1:**
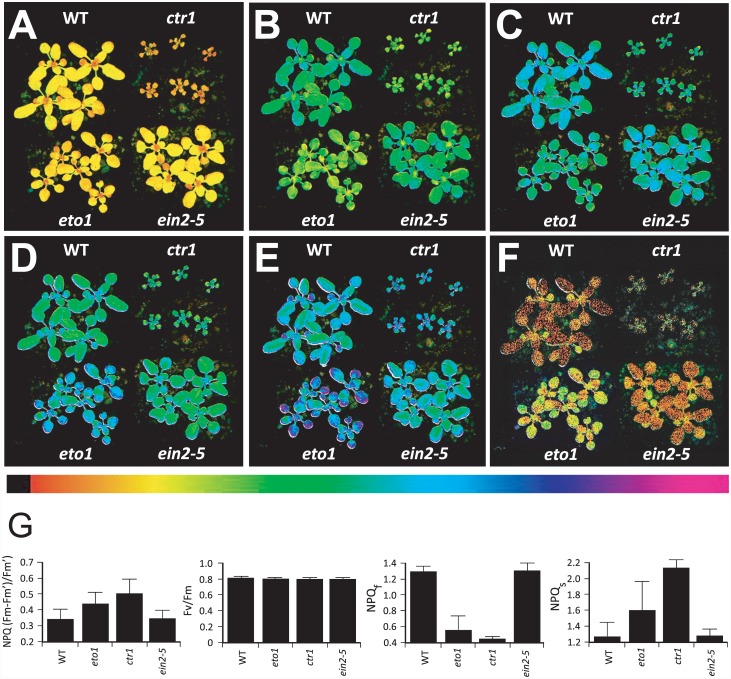
Ethylene regulates induction of NPQ. *eto1-1*, *ctr1-3*, ein2-5, and WT Arabidopsis were grown under 250 PFD for 3.5 weeks. The induction of NPQ in dark-adapted plants exposed to 336 PFD was measured at (A) 20 sec, (B) 60 sec, (C) 2 min, (D) 10 min, (E) 15 min using chlorophyll fluorescence video imaging. (F) Inhibition of PSII quantum yield at 20 min of recovery in the dark following a 25 min light exposure to 336 PFD was measured as 1—(sample F_v_/F_m_/dark-adapted F_v_/F_m_). The level of NPQ induced is presented as false color images according to the color bar below the images. (G) NPQ and F_v_/F_m_ were measured in leaves of 3.5 week-old WT, *eto1-1*, and *ctr1-3* plants grown at 250 PFD following exposure to 400 PFD. Fast and slow relaxation of NPQ (i.e., NPQ_f_ and NPQ_s_, respectively) were measured following exposure to 1800 PFD for 30 min. The data reported are the average of five biological replicates.

NPQ is a light-inducible process that competes for and diverts absorbed energy from photochemistry in order to regulate light harvesting and photosynthetic performance during conditions of excess light [48]. Following exposure of dark-adapted plants to 336 μmol photons m^-2^ s^-1^ (PFD), an aberrant NPQ induction profile was observed in leaves of *eto1-1*, in which ethylene production is elevated, and in *ctr1-3*, in which the ethylene response is constitutive (Fig 1). Although *eto1-1* and *ctr1-3* exhibit reduced plant stature resulting from an ethylene-mediated reduction in cell size [14, 80, 81], NPQ is a ratio of fluorescence values, and therefore, is independent of leaf area or cell size. Thus, comparisons of NPQ among mutant and wild-type (WT) plants can be made without the need for normalization for cell size. During the initial exposure to light, NPQ was lower in *eto1-1* and *ctr1-3* relative to WT and the ethylene insensitive *ein2-5* mutant (Fig 1A and 1B) but its level in *eto1-1* eventually exceeded the WT level under these conditions (Fig 1D and 1E). During recovery in the dark, measurements of the inhibition of PSII quantum yield indicated that *eto1-1* was slower to recover (Fig 1F). Using endpoint measurements in plants with a similar dark-adapted F_v_/F_m_, NPQ was significantly elevated in *ctr1-3* (*p* < 0.05) than in WT leaves following 60 min of exposure to 400 PFD (Fig 1G). Because elevated ethylene production or signaling results in a complex and dynamic alteration of total NPQ accumulation in response to light exposure, whether NPQ in *eto1-1* or *ctr1-3* is observed to be lower or higher than WT depends on when it is measured during its induction (see discussion of S1A Fig below).

NPQ is composed of qE, which serves to dissipate excess absorbed excitation energy as heat, qT, which is quenching associated with state transition, qI or photoinhibitory processes, and qZ, which is independent of PsbS and distinct from state transitions (qT) [47]. The processes that contribute to NPQ can be grouped into those that relax quickly following the transfer of light-treated plants to the dark and those that relax slowly. These can be measured as the fast and slow relaxation components of NPQ (i.e., NPQ_f_ and NPQ_s_, respectively) during the recovery of plants from exposure to high light. NPQ_f_ largely represents qE whereas NPQ_s_ represents slower components of NPQ, including qI. To examine whether the aberrant induction characteristics of NPQ in *eto1-1* and *ctr1-3* are due to a change in the fast or slow relaxation components of NPQ, we measured NPQ_f_ and NPQ_s_. For dark-adapted plants grown at 250 PFD and exposed to 1800 PFD, NPQ_f_ was significantly lower in *eto1-1* (*p* < 0.001) and *ctr1-3* (*p* < 0.0001) relative to WT (Fig 1G), and NPQ_s_ significantly higher in *ctr1-3* (*p* < 0.001) relative to WT (Fig 1G), suggesting an impaired induction of qE that is accompanied by an increase in the slow relaxation component of NPQ.

To determine the kinetics of its induction, we measured NPQ in dark-adapted *eto1-1* and *ctr1-3* plants grown at 250 PFD following exposure of leaves to 400 PFD. NPQ was induced in WT leaves to a high level within 5 min following the imposition of light after which point, NPQ decreased to a steady-state value (S1A Fig). The rapid increase in NPQ immediately following exposure to light is a result of excess light conditions prior to the activation of Calvin cycle reactions. The subsequent decrease in NPQ represents full activation of photochemistry, a process that competes with NPQ for light energy. Following its initial induction and transient decline in *eto1-1* and *ctr1-3*, however, NPQ rose again to levels exceeding the steady-state WT level (S1A Fig). Measuring the induction of NPQ at shorter time intervals to obtain information regarding initial rates revealed a slower initial induction in *eto1-1* and *ctr1-3* which, over time, exceeded the WT level (S1B Fig). The slower initial induction followed by a higher level of NPQ attained in *eto1-1* and *ctr1-3* suggested an impaired induction of qE and an elevated qI, consistent with the observed reduction in NPQ_f_ and increase in NPQ_s_, respectively, in these mutants (Fig 1G). These data reveal that elevated ethylene production or signaling alters total NPQ accumulation in a complex and dynamic manner that results in lower or higher total NPQ accumulation depending on when NPQ is measured during its induction.

A second type of qE quenching, qE_TR_, occurs transiently following the transfer of dark-adapted plants to nonsaturating light [99, 100]. The generation of a transthylakoid membrane proton gradient and PsbS are essential for qE_TR_, but the magnitude of qE_TR_ is also determined by the extent of xanthophyll de-epoxidation [99, 100, 101]. When dark-adapted mutant and WT leaves were exposed to 100 PFD, representing a nonsaturating level of light, NPQ was induced transiently (S1C Fig), representing the light-dependent generation of a transthylakoid membrane pH gradient followed by its breakdown with the activation of Calvin cycle reactions [99, 101, 102]. The maximum initial level of NPQ achieved in *eto1-1* was lower than in WT and was lower still in *ctr1-3*, suggesting a possible reduction in xanthophyll cycle activity and/or PsbS expression.

The elevated ethylene signaling present in *ctr1-3* cannot be reversed using standard inhibitors of ethylene biosynthesis or perception. Therefore, to determine whether inhibiting ethylene signaling prevents the aberrant induction of NPQ in *eto1-1*, we examined the effect of 1-MCP, which blocks ethylene binding to receptors and thus inhibits induction of ethylene responses [103]. Exposure of dark-adapted *eto1-1* plants to 400 PFD resulted in the eventual elevated induction of NPQ relative to WT (S1D Fig) as observed above. Treatment with 1-MCP for 20 hr largely restored a wild-type NPQ induction profile to *eto1-1* and reduced the final level of NPQ achieved in *eto1-1* to a level lower than WT (S1D Fig). Treatment of WT plants with 1-MCP did not significantly alter the kinetics of NPQ induction relative to untreated WT plants. These results support the conclusion that increased ethylene signaling alters the induction of NPQ in response to light.

### Violaxanthin de-epoxidase is repressed by increased ethylene signaling

**Fig 2:**
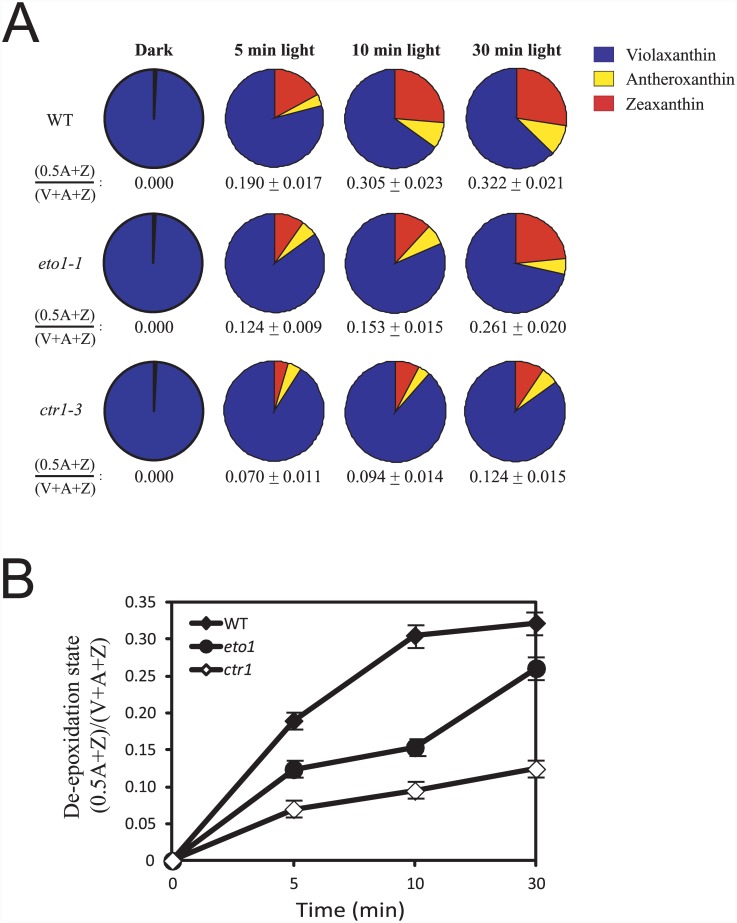
Violaxanthin de-epoxidation is reduced in *eto1-1* and *ctr1-3* under saturating light conditions. (A) Xanthophyll pigments were isolated from leaves of plants (grown at 250 PFD) dark-adapted for 16 hr, or treated with 500 PFD for the times indicated. The pigments were quantitated by HPLC and normalized to chlorophyll a (i.e., μg/mg Chl a). The de-epoxidation status, i.e., (0.5A + Z)/(V + A + Z) was determined from the amounts of violaxanthin (V), antheraxanthin (A), and zeaxanthin (Z) and is included below each pie chart. (B) Graphical display of the de-epoxidation state of the xanthophyll pigments from (A). The data reported are the average of three biological replicates.

The xanthophyll cycle contributes to qE through the light-mediated generation of Z [70, 104]. Exposure to light results in the activation of violaxanthin de-epoxidase (VDE) which catalyzes the de-epoxidation of V to A and Z [105]. As a result, V predominates in dark-adapted plants. Consistent with this, we observed that the predominant xanthophyll cycle pigment in dark-adapted *eto1-1*, *ctr1-3*, and WT leaves was V (Fig 2A). In order to determine whether the aberrant induction of NPQ in *eto1-1* and *ctr1-3* resulted from the reduced de-epoxidation of V, we measured the extent of de-epoxidation in each during exposure to light. Following exposure to 500 PFD, significant de-epoxidation of V to Z was observed in WT leaves within 5 min with additional de-epoxidation occurring upon longer exposure (Fig 2A and 2B). In contrast, the rate of de-epoxidation was significantly lower in *eto1-1*, resulting in a de-epoxidation state that was substantially lower than that in WT (*p* < 0.05, *p* < 0.005, and *p* < 0.05 at 5, 10, and 30 min, respectively) (Fig 2A and 2B). De-epoxidation of V was lower still in *ctr1-3* (*p* < 0.005, *p* < 0.001, and *p* < 0.001 at 5, 10, and 30 min, respectively), resulting in an initial de-epoxidation state that was reduced more than three fold relative to WT (Fig 2A and 2B). The pool size of lutein and neoxanthin was not substantially different among the lines (data not shown). These data suggest that de-epoxidation activity is reduced in *eto1-1* and *ctr1-3*, consistent with their lower initial induction of NPQ (S1B Fig) and lower NPQ_f_ (Fig 1G).

**Fig 3:**
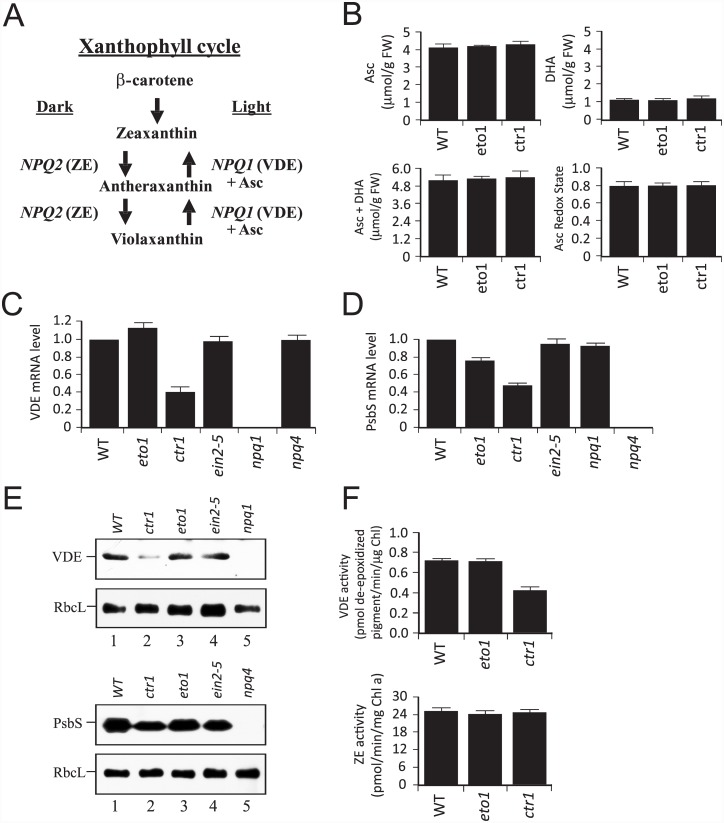
Increased ethylene signaling represses VDE and PsbS expression. (A) The xanthophyll cycle. *NPQ1* encodes violaxanthin de-epoxidase (VDE) whereas *NPQ2* encodes zeaxanthin epoxidase (ZE). (B) The pool sizes for Asc, DHA, total ascorbate (i.e., Asc + DHA), and the Asc redox state were measured in leaves of 4 week-old *eto1-1*, *ctr1-3*, and WT plants. (C) qPCR analysis of *NPQ1* mRNA in leaves of 3 week-old plants. (D) qPCR analysis of PsbS mRNA in leaves of 3 week-old plants. (E) VDE and PsbS protein levels were measured by Western analysis in leaves of 3 week-old plants. Western analysis of the large subunit of Rubisco served as a control. Loading was on an equal chlorophyll basis. (F) VDE and ZE enzyme activity were measured in leaves of 3 week-old *eto1-1*, *ctr1-3*, and WT plants. The data reported are the average and standard deviation of three biological replicates.

VDE, which catalyzes the de-epoxidation reaction, requires ascorbic acid (Asc) as a cofactor (Fig 3A) [106]. To determine whether a reduction in Asc may account for the observed reduction in de-epoxidation activity in *eto1-1* and *ctr1-3*, we measured the levels of Asc and its oxidized form, dehydroascorbate (DHA). The levels of Asc, DHA, and the Asc redox state in *eto1-1* and *ctr1-3* were similar to WT (Fig 3B), suggesting that their reduced de-epoxidation activity was not a result of limited Asc availability.

The reduction in de-epoxidation activity in *eto1-1* and *ctr1-3* could result from a reduction in the expression and/or activation of VDE (Fig 3A). To determine whether the transcript level of VDE was affected in *eto1-1* and *ctr1-3*, we performed qPCR analysis of *NPQ1* mRNA. No expression was detected in the *npq1* mutant (Fig 3C), which lacks VDE expression [49], confirming the specificity of the qPCR analysis. The *npq1* mutant also lacked VDE protein as determined by Western analysis (Fig 3E), confirming the specificity of the antiserum. The level of *NPQ1* mRNA in *ctr1-3* was 52.1% of WT (*p* < 0.01) (Fig 3C), consistent with the lower level of VDE protein in this mutant (Fig 3E). The level of *NPQ1* mRNA (Fig 3C) and VDE protein (Fig 3E) in *eto1-1* was similar to WT, suggesting a high level of ethylene signaling is required for the repression of VDE expression. The level of *NPQ1* mRNA in *ein2-5* was largely unchanged relative to WT (Fig 3C), correlating with a level of VDE protein that was similar to WT (Fig 3E). VDE expression was also unchanged in the *npq4* mutant (Fig 3C), which is null for PsbS [107], demonstrating that VDE expression is not affected by the absence of PsbS expression. We also performed qPCR analysis of PsbS, which is required for qE. The level of PsbS mRNA in *ctr1-3* was 42.0% of WT (*p* < 0.05) (Fig 3D), consistent with the lower level of PsbS protein in the mutant (Fig 3E). A more moderate reduction in PsbS transcript was observed in *eto1-1* but this was not significantly lower than WT (Fig 3D). These results suggest that a high level of ethylene signaling represses VDE and PsbS expression.

To determine whether a reduction in VDE enzyme activity correlated with the transcript and protein data for *eto1-1* and *ctr1-3*, we measured its activity. The assay for VDE involves an *in vitro* pH-mediated activation of its activity, mimicking the light-mediated acidification of the thylakoid lumen where VDE resides. As a consequence, the VDE assay measures the maximum capacity of VDE activity, not necessarily the level of activation achieved *in vivo*, as this depends on the magnitude of the transthylakoid membrane pH gradient established. The maximum level of VDE activity measured in *ctr1-3* was just 43.6% of the WT level (Fig 3F), correlating with the reductions in transcript and protein levels observed for this mutant. The maximum level of VDE activity measured in *eto1-1* was similar to the WT level (Fig 3F), which also correlated with a level of transcript and protein that was similar to WT. The reduced level of de-epoxidation in light-treated *eto1-1* and *ctr1-3* could also have been a result of an increase in zeaxanthin epoxidase (ZE) activity, which catalyzes the epoxidation of Z to A and then to V, i.e., the reverse of VDE activity (Fig 3A). ZE enzyme activity in *eto1-1* and *ctr1-3*, however, was little changed relative to the WT level (Fig 3F), suggesting that the slower rate of de-epoxidation observed in *eto1-1* and *ctr1-3* was not due to altered ZE activity.

### *NPQ1* promoter activity is regulated by ethylene

**Fig 4:**
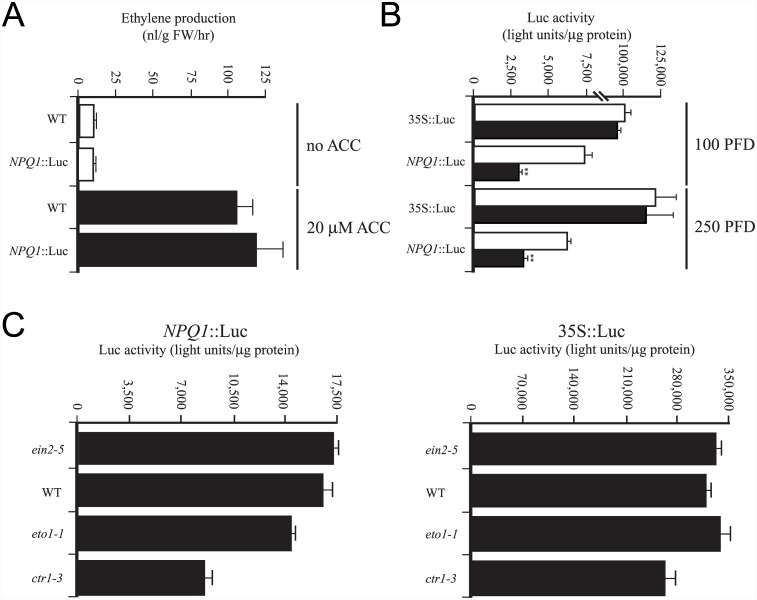
Ethylene represses *NPQ1* promoter activity. (A) Ethylene evolution from WT Arabidopsis and transgenic seedlings containing *NPQ1*::Luc grown for 2 weeks in the absence or presence of 20 μM ACC. (B) Luciferase expression from transgenic Arabidopsis seedlings containing the *NPQ1*::Luc transgene grown at 100 PFD for 2 weeks in the presence of AgNO_3_ (white bars) or 20 μM ACC (black bars). Transgenic Arabidopsis containing a 35S::Luc construct was included as a control under the same growth conditions. The same lines grown at 100 PFD were also transferred to 250 PFD for 24 hr prior to assaying. Bars with asterisks indicate significant difference (*p* < 0.01) relative to the same line grown in the presence of AgNO_3_. (C) Luciferase expression from of 3 week-old transgenic WT, *eto1-1*, *ctr1-3*, and *ein2-5* seedlings containing the *NPQ1*::Luc or 35S::Luc transgenes grown at 250 PFD.

The reduction in *NPQ1* transcript level in *ctr1-3* may result from reduced *NPQ1* promoter activity or increased *NPQ1* mRNA turnover. To distinguish between these two possibilities, we fused the *NPQ1* promoter to the firefly luciferase reporter and, following transformation of wild-type Arabidopsis, we isolated lines homozygous for each construct. We used Arabidopsis containing the 35S promoter driving expression of luciferase as a control. We grew seedlings homozygous for each construct in the presence or absence of ACC, which is converted by the seedlings to ethylene, thus elevating the endogenous production of ethylene (Fig 4A). The level of luciferase activity in 35S::Luc seedlings grown in the presence or absence of ACC was similar (Fig 4B), demonstrating that a 10.6-fold increase in ethylene production does not substantially affect luciferase expression from the 35S promoter. In contrast, an approximate 2-fold reduction in expression in *NPQ1*::Luc seedlings was observed when grown in the presence of ACC relative to growth in the absence of ACC (Fig 4B), suggesting *NPQ1* promoter activity is repressed by elevated ethylene.

We also examined genetically the regulation by ethylene of *NPQ1* promoter activity by introducing the *NPQ1*::Luc construct or the 35S::Luc construct into *eto1-1*, *ctr1-3*, and *ein2*-5 through crosses with wild-type lines containing these constructs. We then isolated progeny homozygous for each Luc construct and the respective ethylene mutation. *NPQ1* promoter activity as measured by luciferase activity was reduced in *eto1-1* and *ctr1-3* to 88.7% and 53.3%, respectively, of the level observed in WT seedlings (Fig 4C). *NPQ1* promoter activity was slightly greater in *ein2-5* than in WT seedlings but this difference was not significant (Fig 4C). Expression from the control 35S::Luc construct in *eto1-1*, *ctr1-3* and *ein2-5* was 104%, 80.9%, and 104% respectively, of that in WT seedlings (Fig 4C). These data indicate that *NPQ1* promoter activity is repressed by an increase in ethylene signaling resulting from increased endogenous ethylene production (e.g., WT seedlings grown in the presence of ACC) or genetically through an increase in ethylene responses (e.g., *eto1-1* and *ctr1-3*). As the reduction in *NPQ1* promoter activity observed in *eto1-1* was modest at best, a significant increase in ethylene signaling is required (e.g., *ctr1-3*) for the repression of *NPQ1* promoter activity to be reflected at the protein (Fig 3E) and enzyme activity levels (Fig 3E). The extent of the repression of *NPQ1* promoter activity observed in *ctr1-3* is consistent with the approximate 2-fold reduction in *NPQ1* transcript (Fig 3C), protein (Fig 3E), and *in vitro* enzyme activity (Fig 3F). The even greater reduction in the rate of de-epoxidation observed *in vivo* for *eto1-1* and *ctr1-3* (Fig 2B), however, raised the possibility that the light-mediated activation of VDE enzyme activity may also be repressed following an increase in ethylene signaling.

### Increased ethylene signaling reduces the light-mediated activation of VDE

Although a modest reduction in *NPQ1* promoter activity was observed in *eto1-1*, the lack of change in *NPQ1* transcript (Fig 3C) or protein (Fig 3E) suggested that the reduction in de-epoxidation observed for this mutant (Fig 2) may be due to a lower degree of activation of VDE in response to light. VDE activity is regulated by the pH of the lumen whereas ZE is constitutively active. VDE undergoes activation during the acidification of thylakoid lumen which is generated by proton pumping from the stroma during photosynthesis, resulting in a lumen that can approach pH 4–5 and a stroma that can approach pH 8 [108]. Stromal pH determines the concentration of total carbonic CO_2_ which is mostly present as HCO_3_^-^ in the basic stroma during photosynthesis [108]. As CO_2_ is the ultimate sink for electrons transported through the photosystems, a reduction in total carbonic CO_2_ in the stroma can cause the overreduction of PSII, leading to an increase in ROS generation and photoinhibition [109].

**Fig 5:**
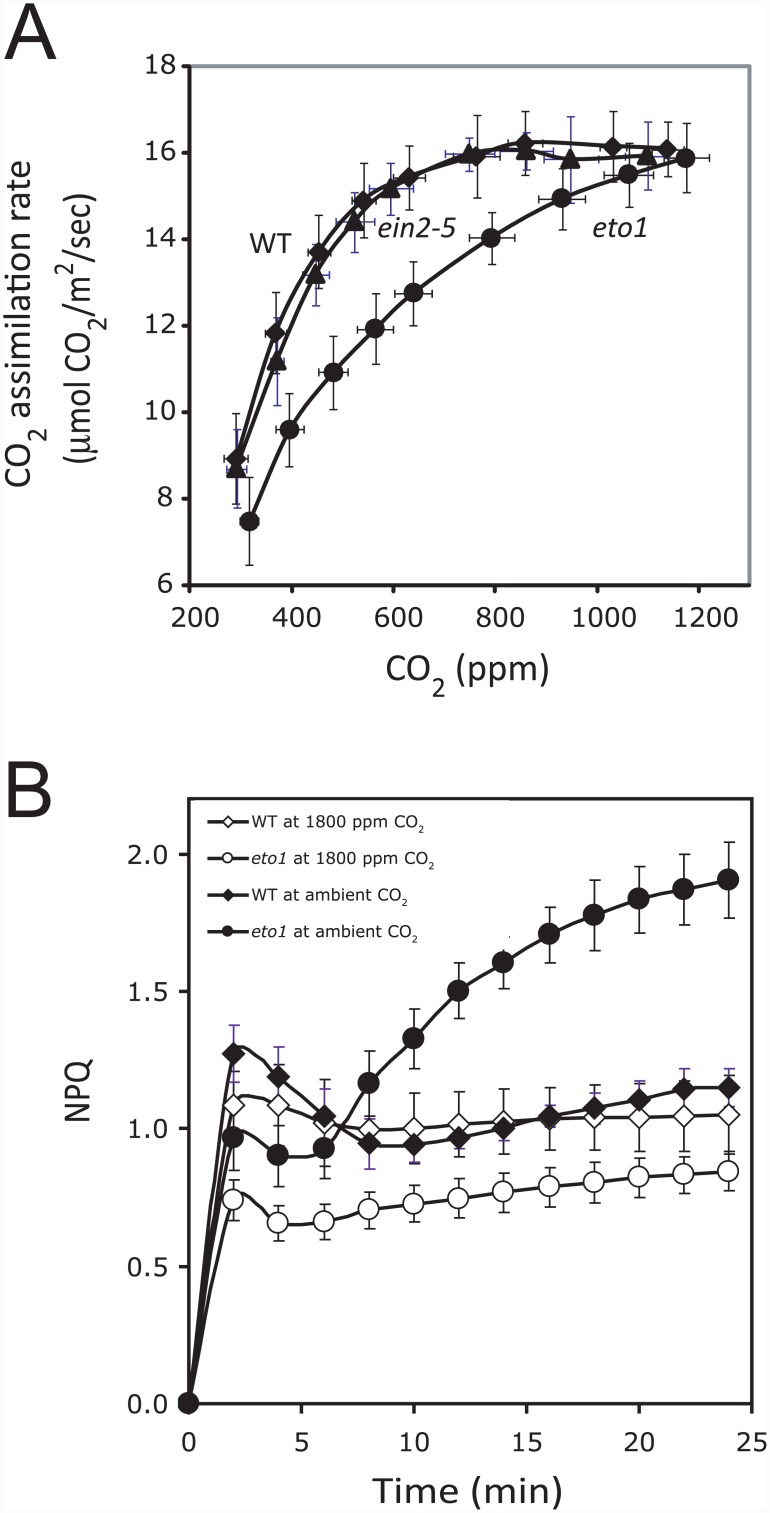
The defect in CO_2_ assimilation in *eto1-1* is corrected by increasing CO_2_ availability. (A) The rate of CO_2_ assimilation was measured in light-adapted WT, *ein2-5*, and *eto1-1* plants at 400 PFD as a function of CO_2_ concentration. The rate of CO_2_ assimilation is plotted against the internal CO_2_ concentration (C_i_). WT (filled diamonds); *ein2-5* (filled triangles); *eto1-1* (filled circles). (B) Induction of NPQ was measured in dark-adapted WT and *eto1-1* plants grown at 250 PFD and exposed to 400 PFD under ambient or 1800 ppm CO_2_. WT (filled diamonds); *eto1-1* (filled circles); WT (open diamonds); and *eto1-1* (open circles).

If the reduction in de-epoxidation observed in *eto1-1* resulted from a reduced transthylakoid membrane pH gradient, this may be accompanied by a lower rate of CO_2_ assimilation and a higher intercellular partial pressure of CO_2_ (C_i_) needed to support a maximum rate of CO_2_ assimilation. At ambient CO_2_, *eto1-1* has a lower rate of CO_2_ assimilation than WT or *ein2-5* (Fig 5A). As neither the stomatal conductance nor the stomatal index, i.e., the density of stomata relative to the epidermal cell number, in *eto1-1* was altered, the C_i_ of *eto1-1* was not reduced relative to WT (data not shown), indicating that CO_2_ diffusion was not limiting. The intercellular partial pressure of CO_2_ required to support the maximum rate of CO_2_ assimilation (V_max_) can be determined from an A-C_i_ or CO_2_ response curve in which the rate of CO_2_ assimilation is measured as a function of C_i_. The rate of CO_2_ assimilation increased in WT and *eto1-1* as the intercellular partial pressure of CO_2_ increased (Fig 5A). The rate of CO_2_ assimilation in *eto1-1* remained substantially lower than WT even as the C_i_ reached 740 μbar, a level at which the maximum rate of CO_2_ assimilation was attained in WT and *ein2-5* (Fig 5A). Only as C_i_ was elevated to approximately 1170 μbar did the rate of CO_2_ assimilation in *eto1-1* begin to plateau (Fig 5A). The difference in the intercellular partial pressure of CO_2_ required to support a ½V_max_ of CO_2_ assimilation in *eto1-1* and WT supports the notion of a lower solubility of CO_2_ in the *eto1-1* stroma due to a less basic stroma.

The relative difference in stromal pH in *eto1-1* versus WT leaves can be calculated from the difference in the intercellular partial pressure of CO_2_ required to support a ½V_max_ rate of CO_2_ assimilation. The concentration of CO_2_ dissolved in the stroma, i.e., CO_2_(aq), is determined by CO_2_(aq) = CO_2_(i) x K_H_, where K_H_ is the Henry’s constant for CO_2_ (at 25°C, K_H_ = 3.4x10^-2^ M/atm). Thus, as C_i_ at ½V_max_ is 234 ppm in WT and 326 ppm in *eto1-1*, the concentration of dissolved CO_2_ needed to reach the K_m(CO2)_ for Rubisco is 7.91 x 10^-6^ M in WT and 11.0 x 10^-6^ M in the *eto1-1* stroma. Once dissolved in water, CO_2_ is rapidly converted into carbonic acid (H_2_CO_3_) by carbonate anhydrase and dissociates into HCO_3_^-^ and CO_3_^2-^, which collectively comprises the total stromal carbonic CO_2_, i.e., Total[CO_2_(aq)]. The dissociation equations of H_2_CO_3_ and HCO_3_^-^ and their respective K_a_ values yields: [H^+^]^2^/([H^+^]^2^+[H^+^]*K_a1_+K _a1_*K_a2_) = [CO_2_(aq)]/Total[CO_2_(aq)]. Because K_a1_*K_a2_<<([H^+^]^2^+[H^+^]*K_a1_), the equation can be simplified to: [H^+^]^2^/([H^+^]^2^+[H^+^]*K_a1_) ≈ [CO_2_(aq)]/Total[CO_2_(aq)] or [H^+^] = CO_2_(aq)*K_a1_/(Total[CO_2_(aq)]-CO_2_(aq)). The relative difference in stromal pH in *eto1-1* versus WT leaves can be determined from:

$$\frac{{\left[{\text{H}}^{\text{+}}\right]}_{\text{wt}}}{{\left[{\text{H}}^{\text{+}}\right]}_{eto1-1}}\;=\;\frac{{\text{CO}}_{\text{2}}{\left(\text{aq}\right)}_{\text{wt}}{\text{*K}}_{\text{a1}}\text{/}\left(\text{Total}{\left[{\text{CO}}_{\text{2}}\left(\text{aq}\right)\right]}_{\text{wt}}{\text{-CO}}_{\text{2}}{\left(\text{aq}\right)}_{\text{wt}}\right)}{{\text{CO}}_{\text{2}}{\left(\text{aq}\right)}_{eto1-1}{\text{*K}}_{\text{a1}}\text{/}\left(\text{Total}{\left[{\text{CO}}_{\text{2}}\left(\text{aq}\right)\right]}_{eto1-1}{\text{-CO}}_{\text{2}}{\left(\text{aq}\right)}_{eto1-1}\right)}$$

Because Total[CO_2_(aq)]>>CO_2_(aq) and Total[CO_2_(aq)] is equivalent in *eto1-1* and WT at ½ V_max_, the equation can be simplified to: [H^+^]_wt_/[H^+^]_*eto1-1*_ ≈ CO_2_(aq)_wt_/ CO_2_(aq)_*eto1-1*_ or pH_wt_-pH_*eto1-1*_ ≈ lg(CO_2_(aq)_*eto1-1*_)- lg(CO_2_(aq)_wt_) = lg(11.0x10^-6^) − lg(7.91x10^-6^) = 0.142. Therefore, if the pH in the WT stroma is 7.8 during photosynthesis, it would be approximately 7.658 in the *eto1-1* stroma following light exposure. The observation that the level of carbonic anhydrase activity was actually elevated in *eto1-1* (and *ctr1-3*), particularly in chloroplasts, when plants were grown at 250 PFD or in sunlight (Table 1) demonstrated that a decrease in carbonic anhydrase activity in *eto1-1* was not responsible for a reduction in stromal carbonic CO_2_. The increase in carbonic anhydrase activity in *eto1-1*, however, may represent an attempt to compensate for its lower stromal pH.

**Table 1:** Carbonic anhydrase activity in ethylene mutants.

	Carbonic anhydrase activity^a^ (unit activity/mg protein)			
	Total cell	t-test	Chloroplast fraction	t-test
WT	143 ± 16.2		41.4 ± 2.8	
*eto1*	191 ± 26.7	P<0.05	121.4 ± 8.2	P<0.001
*ctr1*	155 ± 17.9	P = 0.356	79.0 ± 6.5	P<0.001

^a^Determined from three replicates grown at 250 PFD for three weeks. The average and standard deviation for each are reported.

The lower stromal pH in *eto1-1* suggests a reduced ability to establish and/or maintain a proton gradient across the thylakoid membrane. To confirm directly if the transthylakoid membrane pH gradient (ΔpH) in *eto1-1* and *ctr1-3* is smaller than in WT, we measured the ΔpH in intact chloroplasts by measuring the fluorescence of 9-aminoacridine (9-AA) which is quenched as a function of the light-mediated decrease in pH in the thylakoid lumen [110, 111, 112, 113, 114]. The relative difference in the ΔpH was significantly smaller in *eto1-1* and *ctr1-3* than in WT following light exposure (Table 2). In contrast, the relative difference in the ΔpH in *npq1* or *npq4* was not significantly different from WT. Addition of 2,6-dichloro-*p*-benzoquinone (DCBQ), an exogenous PSII electron acceptor that enables maximum electron flow from PSII reaction centers, increased the ΔpH established upon exposure to light as expected but *eto1-1* and *ctr1-3* continued to exhibit a smaller relative increase in the ΔpH than WT (Table 2). The inability to fully acidify the thylakoid lumen in *eto1-1* would limit the light-mediated activation of its VDE. This conclusion is consistent with the approximate 2-fold reduction in de-epoxidation activity in *eto1-1* within the initial 10 min of light exposure (Fig 2B) and the observation that increasing C_i_ disproportionately increases the level of photochemistry in *eto1-1* relative to WT (Fig 5A). Although VDE expression in *ctr1-3* was reduced approximately 2-fold (Fig 3), the 4-fold reduction in its de-epoxidation activity within the initial 10 min of light exposure (Fig 2B) and the substantially reduced transthylakoid membrane pH gradient during light exposure (Table 2) also indicates a reduced activation of VDE in this mutant.

**Table 2:** The transthylakoid membrane pH gradient is reduced in ethylene mutants.

	No DCBQ			0.2 mM DCBQ		
	ΔpH of Transthylakoid Membrane^a^	t-test	Relative Transthylakoid Membrane ΔpH	ΔpH of Transthylakoid Membrane^a^	t-test	Relative Transthylakoid Membrane ΔpH
WT	2.783		1.000	3.103		1.000
*eto1*	2.700	P<0.001	0.827	2.995	P<0.005	0.779
*ctr1*	2.677	P<0.001	0.784	2.956	P<0.001	0.712
*npq1*	2.759	P = 0.116	0.946	3.095	P = 0.682	0.982
*npq4*	2.788	P = 0.107	1.012	3.095	P = 0.686	0.982

^a^Determined from plants grown at 250 PFD for three weeks that were exposed to 800 PFD for 8 min.

The average of three replicates and standard deviation for each are reported.

Although the reduced VDE activity in *eto1-1* can account for the slower initial rate of induction of NPQ (S1B Fig), it does not account for the gradual increase in NPQ that eventually overtakes the WT steady-state level (S1 Fig). Reductions in the transthylakoid membrane pH gradient and stromal CO_2_ levels could result in increased ROS and photoinhibitory processes that would be measured as the qI component of NPQ as indicated by the increase in NPQ_s_ (Fig 1G). If so, increasing the level of CO_2_ dissolved in the *eto1-1* stroma by increasing the C_i_ should reduce qI which would be indicated by the absence of the gradual increase in NPQ that overtakes the WT steady-state level. To test this, we measured the induction of NPQ in *eto1-1* leaves exposed to 1800 ppm CO_2_ in order to saturate the *eto1-1* stroma. The induction of NPQ in *eto1-1* at ambient CO_2_ exhibited the same increase in NPQ that overtakes the WT steady-state level (Fig 5B) as observed above (S1A Fig). At 1800 ppm CO_2_, however, the elevated induction of NPQ in *eto1-1* was reversed (Fig 5B). These results suggest that increasing the level of soluble CO_2_ in the *eto1-1* stroma is sufficient to prevent the aberrant accumulation of NPQ following prolonged exposure to high light, consistent with the conclusion that the elevated increase of NPQ in *eto1-1* represents photoinhibition.

### Increasing VDE expression reverses the defect in NPQ in *eto1-1* and *ctr1-3*

**Fig 6:**
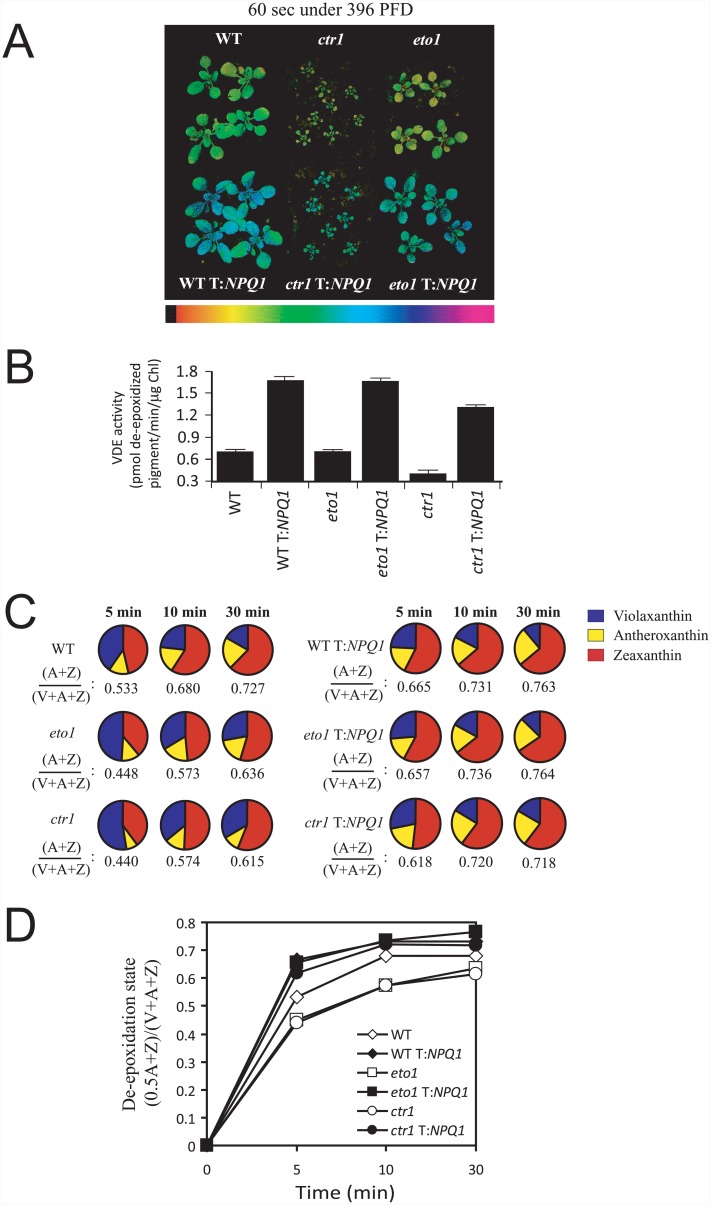
Restoring VDE expression corrects the aberrant NPQ induction and violaxanthin de-epoxidation in *eto1-1* and *ctr1-3* plants. (A) The induction of NPQ in dark-adapted WT, *eto1-1*, and *ctr1*-3 plants with or without the 35S::*NPQ1* transgene following their exposure to 396 PFD for 60 sec using chlorophyll fluorescence video imaging. The level of NPQ is presented as false color images as indicated by the color bar below the image. Dark-adapted *eto1-1*, *ctr1-3*, and WT (grown at 250 PFD) without (B) or with (C) the 35S::*NPQ1* transgene were treated with 1000 PFD for the times indicated. Xanthophyll pigments were quantitated by HPLC and normalized to chlorophyll a (i.e., μg/mg Chl a). (D) The kinetics of the rate of de-epoxidation is shown.

The above results suggest that elevated ethylene signaling represses VDE activation and, at very high levels, represses VDE expression as well. To investigate whether restoring VDE activity in *eto1-1* or *ctr1-3* would correct the impaired functioning of the xanthophyll cycle and the induction of NPQ, we used a transgenic approach to increase the expression of VDE in these mutants. For this, the Arabidopsis *NPQ1* coding region was placed under the control of the 35S promoter, which is largely unaffected by ethylene (Fig 4A), and the construct introduced into wild-type Arabidopsis. We screened transformants containing the construct for expression using Western analysis and identified a VDE overexpressing line (WT T::*NPQ1*) exhibiting elevated qE [115]. We introduced the 35S::*NPQ1* transgene into *eto1-1* or *ctr1-3* through crosses and isolated the homozygous *eto1-1* T::*NPQ1* and *ctr1-3* T::*NPQ1* lines. WT, *eto1-1*, and *ctr1-3* containing the *NPQ1* transgene exhibited a greater induction of NPQ (Fig 6A) which correlated with a higher level of *in vitro* VDE activity (Fig 6B).

To examine whether increasing VDE activity in *eto1-1* and *ctr1-3* corrected the defect in the functioning of the xanthophyll cycle, we measured the generation of A and Z in response to high light (i.e., 1000 PFD) in the mutants containing the 35S::*NPQ1* transgene. As was observed in Fig 2, the generation of A and Z was substantially lower in *eto1-1* and *ctr1-3* than WT (Fig 6C and 6D), despite the higher level of light used relative to that in Fig 2. Increasing VDE expression in *eto1-1* and *ctr1-3* increased the generation of A and Z (Fig 6C) and increased the rate of de-epoxidation to a level similar to WT plants containing the 35S::*NPQ1* transgene (Fig 6D). Increasing VDE activity did not alter zeaxanthin epoxidase activity or substantially alter the pool sizes of neoxanthin or lutein (data not shown).

To determine whether increasing VDE activity in *eto1-1* or *ctr1-3* corrected their aberrant NPQ induction profile, we measured the kinetics of NPQ induction. During exposure to saturating light, i.e., 1000 PFD, the initial rate of NPQ induction was lower in *eto1-1* and *ctr1-3* relative to WT (S2 Fig) as observed above (S1B Fig) but that increasing VDE expression increased the rate of NPQ induction in these mutants (S2A and S2B Fig). Under 100 PFD, NPQ was transiently induced to a lower level in *eto1-1* and *ctr1-3* than in WT but increasing VDE expression in these mutants increased NPQ induction as it did in WT plants (S2C Fig). Similar results were obtained when plants were exposed to 400 PFD, in which an elevated induction of NPQ was observed in *eto1-1* and *ctr1-3* overexpressing VDE (S2D Fig).

### Restoring VDE activity reverses the elevated ROS production and photoinhibition in *eto1-1* and *ctr1-3*

Reduced function of the xanthophyll cycle can result in increased superoxide anion (O_2_^.-^) production as a consequence of the over reduction of the photosystems [44, 116]. To examine whether correcting the function of the xanthophyll cycle in *eto1-1* and *ctr1-3* would reduce ROS levels following exposure to high light, we measured the level of O_2_^.-^ in *eto1-1* and *ctr1-3* overexpressing VDE and compared this to *eto1-1* and *ctr1-3* during exposure to sunlight. The level O_2_^.-^ was substantially higher in *eto1-1* and *ctr1-3* than in WT. It should be noted that the level O_2_^.-^ in *eto1-1* and *ctr1-3* was also higher than in *npq1* or *npq4* (Table 3), indicating that the reduction in VDE expression and activity in *eto1-1* and *ctr1-3* is not solely responsible for the increase in ROS. The restoration of VDE activity in *eto1-1* and *ctr1-3* did reduce the level of O_2_^.-^ generated in response to high light although the levels remained higher than in WT (Table 3). In contrast, overexpression of VDE in WT had little effect on the low level of O_2_^.-^ generated.

**Table 3:** Restoring VDE expression to *eto1-1* and *ctr1-3* reduces the rate of O_2_^.-^ production.

	Superoxide production^a^ (nmol/g FW/min)			
	1900 PFD	t-test	100 PFD	t-test
WT	121 ± 11.6		118 ± 14.1	
WT *T*:*NPQ1*	123 ± 17.4	P = 0.802	119 ± 16.2	P = 0.919
*eto1*	224 ± 11.6	P<0.001	112 ± 13.9	P = 0.760
*eto1 T*:*NPQ1*	187 ± 8.7	P<0.005	140 ± 14.6	P = 0.173
*ctr1*	252 ± 13.3	P<0.001	123 ± 14.7	P = 0.704
*ctr1 T*:*NPQ1*	209 ± 17.6	P<0.005	114 ± 10.6	P = 0.831
*npq1*	177 ± 9.2	P<0.005		
*npq4*	188 ± 3.0	P<0.005		

^a^Determined from three replicates from plants grown at 250 PFD for three weeks that were exposed to the light intensity indicated up to 1 hr. The average and standard deviation for each are reported.

As *eto1-1* and *ctr1-3* exhibited a lower transthylakoid membrane pH gradient during exposure to light, we examined whether restoring VDE activity would correct the impaired transthylakoid membrane pH gradient in *eto1-1* and *ctr1-3* by measuring the quenching of 9-AA in intact chloroplasts. The relative difference in the transthylakoid membrane ΔpH was significantly lower in *eto1-1* and *ctr1-3* than in WT following light exposure (Table 4) in good agreement with the measurements in Table 2. Restoration of VDE activity in *eto1-1* and *ctr1-3* increased the relative difference in the transthylakoid membrane ΔpH generated in response to light exposure. In contrast, overexpression of VDE in the WT background did not increase its relative difference in the transthylakoid membrane ΔpH. When exposed to a subsaturating level of light (i.e., 150 PFD for 3 min), the relative difference in the ΔpH in *eto1-1* was not significantly different from WT but it was lower in *ctr1-3*, although to a smaller extent than following exposure to 800 PFD (Table 4). Restoration of VDE activity in *ctr1-3* restored the relative difference in the ΔpH to the WT level following exposure to low light whereas overexpression of VDE in WT had the opposite effect and reduced the relative difference in the ΔpH (Table 4). These results support the conclusion that *eto1-1* and *ctr1-3* are impaired in their ability to establish or maintain a full ΔpH in high light which can be partially corrected by increasing VDE activity.

**Table 4:** Restoring VDE expression to *eto1-1* and *ctr1-3* increases the transthylakoid membrane pH gradient.

	800 PFD			150 PFD		
	ΔpH of Transthylakoid Membrane^a^	t-test	Relative Transthylakoid Membrane ΔpH	ΔpH of Transthylakoid Membrane^a^	t-test	Relative Transthylakoid Membrane ΔpH
WT	2.759		1.000	2.361		1.000
WT *T*:*NPQ1*	2.746	P = 0.145	0.972	2.263	P<0.001	0.799
*eto1*	2.674	P<0.05	0.823	2.373	P = 0.172	1.030
*eto1 T*:*NPQ1*	2.706	P<0.05	0.887	2.401	P = 0.263	1.097
*ctr1*	2.619	P<0.05	0.725	2.313	P<0.01	0.897
*ctr1 T*:*NPQ1*	2.680	P<0.001	0.835	2.374	P = 0.336	1.031

^a^Determined from plants grown at 250 PFD for three weeks that were exposed to the light intensity indicated for 3 min. The average of three replicates and standard deviation for each are reported.

The impairment in establishing a full transthylakoid membrane pH gradient in *eto1-1* and *ctr1-3* following exposure to high light could be a result of fewer electrons entering PSII reaction centers while the elevated production of O_2_^.-^ in these mutants may indicate an increase in the fraction of those electrons that do enter PSII reaction being used in ROS generation rather than for CO_2_ fixation. To examine whether an increase in ethylene signaling alters electron flow, we measured the electron transport rate (ETR) as a function of increasing light intensity. As would be expected, the ETR increased in WT plants with increasing light (S3A Fig). Interestingly, ETR levels in *eto1-1* and *ctr1-3* were actually higher than in WT. In contrast, the level of ETR in *npq1* and *npq4* was not higher than the WT level. As qE is impaired in *npq1* and *npq4*, this suggests that the increase in ETR in *eto1-1* and *ctr1-3* is not a result of their reduced qE alone. Restoring VDE activity in *eto1-1* and *ctr1-3*, however, decreased the ETR in these mutants as it did in WT (S3B Fig), demonstrating that increasing VDE expression serves to reduce the ETR. These data show that, rather than a reduction in the rate of electron transport, *eto1-1* and *ctr1-3* exhibit an increase in electron transport relative to wild-type plants.

We next measured the quantum requirement of *eto1-1* and *ctr1-3*, i.e., the number of electrons required to fix one molecule of CO_2_, from the relationship between the quantum yield of PSII electron transport (φPSII) and the quantum yield of CO_2_ assimilation (φCO_2_) [117]. A quantum requirement of approximately eight electrons per CO_2_ molecule fixed was observed in WT plants and overexpression of VDE did not alter this significantly (Table 5). In contrast, a quantum requirement of approximately 10.31 and 12.85 electrons per CO_2_ molecule fixed was observed in *eto1-1* and *ctr1-3*, respectively. The restoration of VDE activity in *eto1-1* decreased this elevated quantum requirement significantly (P<0.05 relative to *eto1-1*) as it did in *ctr1-3* (P<0.01 relative to *ctr1-3*) (Table 5) although not to WT levels. These data suggest that the reduced VDE activity of *eto1-1* and *ctr1-3* results in increases in ETR and the quantum requirement and correlates with an increase in O_2_^.-^ production, supporting the notion that a greater number of electrons entering PSII are used to generate ROS. The data also show that restoring VDE activity in *eto1-1* and *ctr1-3* partially reverses their elevated ETR and quantum requirement.

**Table 5:** Increased ethylene signaling increases the electron requirement per molecule CO_2_ fixed.

	Electron Requirement per CO_2_ Fixed	t-test
WT	8.37 ± 0.42	
WT *T*:*NPQ1*	7.95 ± 0.38	P = 0.162
*eto1*	10.31 ± 0.40	P<0.001
*eto1 T*:*NPQ1*	9.30 ± 0.34	P<0.05
*ctr1*	12.85 ± 0.51	P<0.001
*ctr1 T*:*NPQ1*	11.27 ± 0.42	P<0.001

^a^Determined under 2% oxygen from three replicates grown at 250 PFD for three weeks. The average and standard deviation for each are reported.

**Fig 7:**
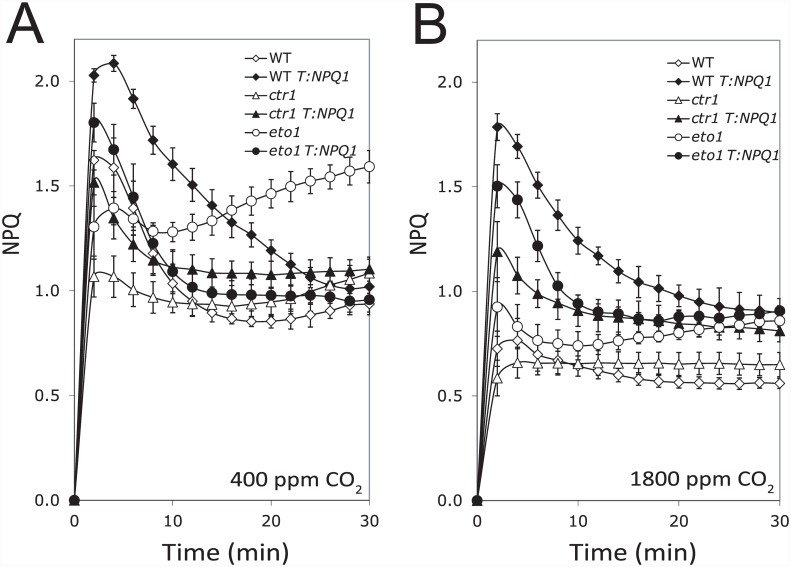
Restoring VDE expression or elevated CO_2_ reverses the qI component of NPQ in *eto1-1* and *ctr1-3*. The aberrant accumulation of NPQ in *eto1-1* leaves is corrected by increasing CO_2_. Induction of NPQ was measured in dark-adapted WT, *eto1-1*, and *ctr1-3* plants grown at 250 PFD and exposed to 400 PFD under (A) ambient or (B) elevated CO_2_ (i.e., 1800 ppm CO_2_). WT (open diamonds); WT T:*NPQ1* (filled diamonds); *eto1-1* (open circles); *eto1-1* T:*NPQ1* (filled circles); and *ctr1*-3 (open triangles); and *ctr1*-3 T:*NPQ1* (filled triangles). The data reported are the average of four replicates.

As restoring VDE activity in *eto1-1* and *ctr1-3* partially reverses the elevated O_2_^.-^ production in these mutants, we examined whether it would also reduce the extent of photoinhibition when exposed to high light. Therefore, the induction of NPQ was measured in the mutants with or without the T::*NPQ1* transgene under ambient and elevated CO_2_ (i.e., 1800 ppm). The induction of NPQ in *eto1-1* and *ctr1-3* under ambient CO_2_ was induced initially to a lower level than in WT (within 2–4 min of light exposure) but eventually overtook the WT steady-state level (Fig 7A) as observed above (Fig 5B). Exposure to 1800 ppm CO_2_, however, substantially reduced this gradual increase in NPQ in *eto1-1* and *ctr1-3* such that following its initial induction, the level NPQ continued to rise only slightly (Fig 7B). Increasing VDE expression in *eto1-1* and *ctr1-3* resulted in a NPQ induction profile under ambient and elevated CO_2_ that was largely similar to WT in that, following its initial induction, NPQ partially relaxed and did not exhibit a subsequent increase above the level observed for WT overexpressing VDE (Fig 7A and 7B, respectively). These results suggest that the qI generated in *eto1-1* and *ctr1-3* can be prevented by restoring VDE activity or by increasing CO_2_.

**Fig 8:**
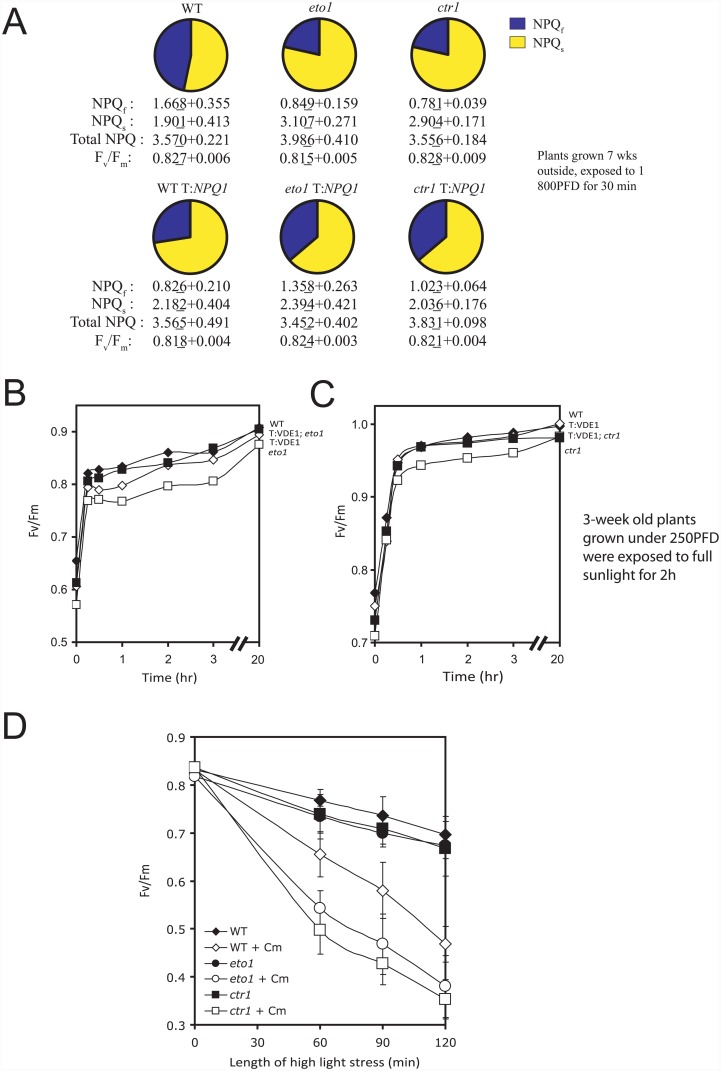
Restoring VDE expression reverses the aberrant relaxation of NPQ in *eto1-1* and *ctr1-3* following exposure to high-light stress. (A) Fast and slow relaxation of NPQ (i.e., NPQ_f_ and NPQ_s_, respectively) were measured in adult leaves of *eto1-1* and *ctr1-3* plants, without or with the 35S::*NPQ1* transgene, following exposure to 1800 PFD for 30 min. WT plants without or with the 35S::*NPQ1* transgene were included in each analysis. Measurements were made prior to the appearance of the inflorescence. Values for each are presented below each pie chart as is the dark-adapted F_v_/F_m_. Values were determined from four replicates. The recovery of (B) *eto1-1* and *eto1-1* T:*NPQ1* plants or (C) *ctr1-3* and *ctr1-3* T:*NPQ1* plants from a 2 hr exposure to sunlight (i.e., 1900 PFD) was determined by measuring F_v_/F_m_ over time following the transfer of plants to darkness to facilitate recovery. The data was expressed relative to dark-adapted F_v_/F_m_ value in order to make direct comparisons between lines. WT (filled diamonds); WT T:*NPQ1* (open diamonds); *eto1-1* and *ctr1*-3 (open squares); *eto1-1* T:*NPQ1* and *ctr1*-3 T:*NPQ1* (filled squares). The data reported are the average of six replicates.

As shown above, *eto1-1* and *ctr1-3* exhibit a smaller NPQ_f_ and larger NPQ_s_, the fast and slow relaxation components of NPQ, respectively (Fig 1G). To examine whether restoring VDE activity in *eto1-1* and *ctr1-3* corrects their defects in NPQ_f_ and NPQ_s_, we measured the fast and slow relaxation components of NPQ in *eto1-1* and *ctr1-3* overexpressing VDE. In dark-adapted plants exposed to 1800 PFD, NPQ_f_ was substantially lower and NPQ_s_ substantially higher in *eto1-1* and *ctr1-3* than in WT (Fig 8A), confirming the photosensitivity of *eto1-1* and *ctr1-3*. A decrease in NPQ_f_ and slight increase in NPQ_s_ was observed in leaves of WT overexpressing VDE (Fig 8A) as previously reported [115]. In contrast, NPQ_f_ was significantly increased and NPQ_s_ significantly reduced in *eto1-1* T::*NPQ1* and *ctr1-3* T::*NPQ1* relative to *eto1-1* and *ctr1-3* (Fig 8A), consistent with the effect that overexpressing VDE had on reducing the qI component of NPQ in *eto1-1* and *ctr1-3* (Fig 7). As increasing VDE expression in WT plants did not have the same effect on NPQ_f_ and NPQ_s_, these results suggest its effect was specific to these mutants.

To examine whether the restoration of VDE activity in *eto1-1* and *ctr1-3* protects against damage to the PSII reaction center, we measured the recovery of *eto1-1* and *eto1-1* T:*NPQ1* plants following a 2 hr exposure to sunlight (i.e., 1900 PFD) by measuring the quantum yield of PSII (i.e., F_v_/F_m_), upon their transfer to darkness. The data was expressed relative to the dark-adapted F_v_/F_m_ value (set to a value of 1) in order to make direct comparisons between lines. High light treatment of dark-adapted plants resulted in a substantial drop in their relative F_v_/F_m_ from which they largely recovered during the subsequent 20 hr in dark (Fig 8B and 8C). *eto1-1* and *ctr1-3* exhibited a greater reduction in the relative F_v_/F_m_ following high light treatment and a slower rate of recovery than did WT plants (Fig 8B and 8C). Overexpression of VDE did not improve the rate of recovery in WT plants but did improve the rate of recovery in *eto1-1* and *ctr1-3* (Fig 8B and 8C). The observation that increasing VDE activity in WT plants does not increase phototolerance under these conditions supports the conclusion that the ability of restoring VDE activity to increase phototolerance in *eto1-1* and *ctr1-3* is specific to these mutants.

Recovery from photoinhibition requires new protein synthesis to repair damaged PSII reaction centers [118, 119, 120, 121, 122]. Inhibiting this repair with inhibitors of chloroplast protein synthesis results in a greater reduction in the quantum efficiency (i.e., F_v_/F_m_) during exposure to light [123]. To examine whether the rate of photoinhibition in *eto1-1* and *ctr1-3* was greater than in WT in the absence of repair activity, we infiltrated adult leaves with either 1 mM chloramphenicol/0.1% ethanol, which inhibits chloroplast protein synthesis, or 0.1% ethanol only and exposed to 1000 PFD. We then measured the quantum yield of PSII (i.e., F_v_/F_m_) during light exposure. In the absence of chloramphenicol, the quantum yield of *eto1-1* and *ctr1-3* decreased at a greater rate than in WT (Fig 8D). The reduction in the quantum yield of all lines was greater in the presence of chloramphenicol than it was in its absence but the decrease in *eto1-1* and *ctr1-3* was even greater than the WT rate (Fig 8D), suggesting that *eto1-1* and *ctr1-3* experience a greater level of photodamage particularly when the repair of PSII reaction centers is inhibited.

As α-tocopherol also protects against photodamage, we examined whether the greater photosensitivity of *eto1-1* and *ctr1-3* may be due to reduced level of α-tocopherol. We measured α-tocopherol content in plants grown at 250 PFD and exposed to sunlight up to 6 hr. The level of α-tocopherol was not significantly different in *eto1-1* relative to the WT prior to and following sunlight exposure (Table 6). α-Tocopherol content in *ctr1-3* was actually higher than in WT plants both before and after exposure to sunlight. Restoring VDE expression in *eto1-1* and *ctr1-3* did not alter the level of α-tocopherol in WT or mutants plants, indicating that the greater photosensitivity of *eto1-1* and *ctr1-3* is not a result of a reduction in α-tocopherol levels. α-Tocopherol content in *npq1* was also higher than in WT plants (Table 6) and, as previously reported, the higher levels of α-tocopherol and ascorbic acid in *npq1* may have protected against ROS-mediated lipid peroxidation during exposure to high light [124]. As *eto1-1* exhibited no increase in α-tocopherol or ascorbic acid content (Table 6 and Fig 3), its reduced VDE activity occurs in the absence of any increase in these compensating antioxidants. Moreover, although *ctr1-3* exhibited an increase in α-tocopherol content, it showed no increase in ascorbic acid content (Fig 3).

**Table 6:** Restoring VDE expression in *eto1-1* and *ctr1-3* does not alter α-tocopherol content.

	α-Tocopherol^a^ (nmol/g FW)							
	0 hr sunlight	t-test	1 hr sunlight	t-test	3 hr sunlight	t-test	6 hr sunlight	t-test
WT	9.00 ± 0.77		10.3 ± 0.9		10.4 ± 0.3		10.9 ± 0.3	
WT *T*:*NPQ1*	9.44 ± 0.78	P = 0.596	10.4 ± 0.6	P = 0.911	11.0 ± 0.5	P = 0.192	11.5 ± 0.6	P = 0.246
*eto1*	8.82 ± 0.44	P = 0.790	10.6 ± 0.5	P = 0.755	11.4 ± 0.3	P<0.05	11.0 ± 0.8	P = 0.824
*eto1 T*:*NPQ1*	9.12 ± 0.95	P = 0.889	10.3 ± 0.4	P = 0.936	11.2 ± 0.	P = 0.172	11.6 ± 0.7	P = 0.255
*ctr1*	12.31 ± 0.59	P<0.01	14.2 ± 0.9	P<0.05	14.1 ± 0.7	P<0.01	14.5 ± 0.8	P<0.05
*ctr1 T*:*NPQ1*	12.01 ± 0.64	P<0.05	14.4 ± 0.7	P<0.01	14.2 ± 0.8	P<0.05	14.6 ± 0.9	P<0.05
*npq1*	11.86 ± 0.34	P<0.05	13.1 ± 0.7	P<0.05	13.6 ± 0.8	P<0.05	14.1 ± 1.1	P<0.05
*npq4*	9.79 ± 0.49	P = 0.298	10.3 ± 0.6	P = 0.984	10.7 ± 0.5	P = 0.554	10.9 ± 0.4	P = 0.840

^a^Determined from three replicates from plants grown at 250 PFD for three weeks that were exposed to sunlight for the time indicated. The average and standard deviation for each are reported.

### Restoring VDE activity reverses the small growth phenotype imposed by a moderate increase in ethylene signaling

**Fig 9:**
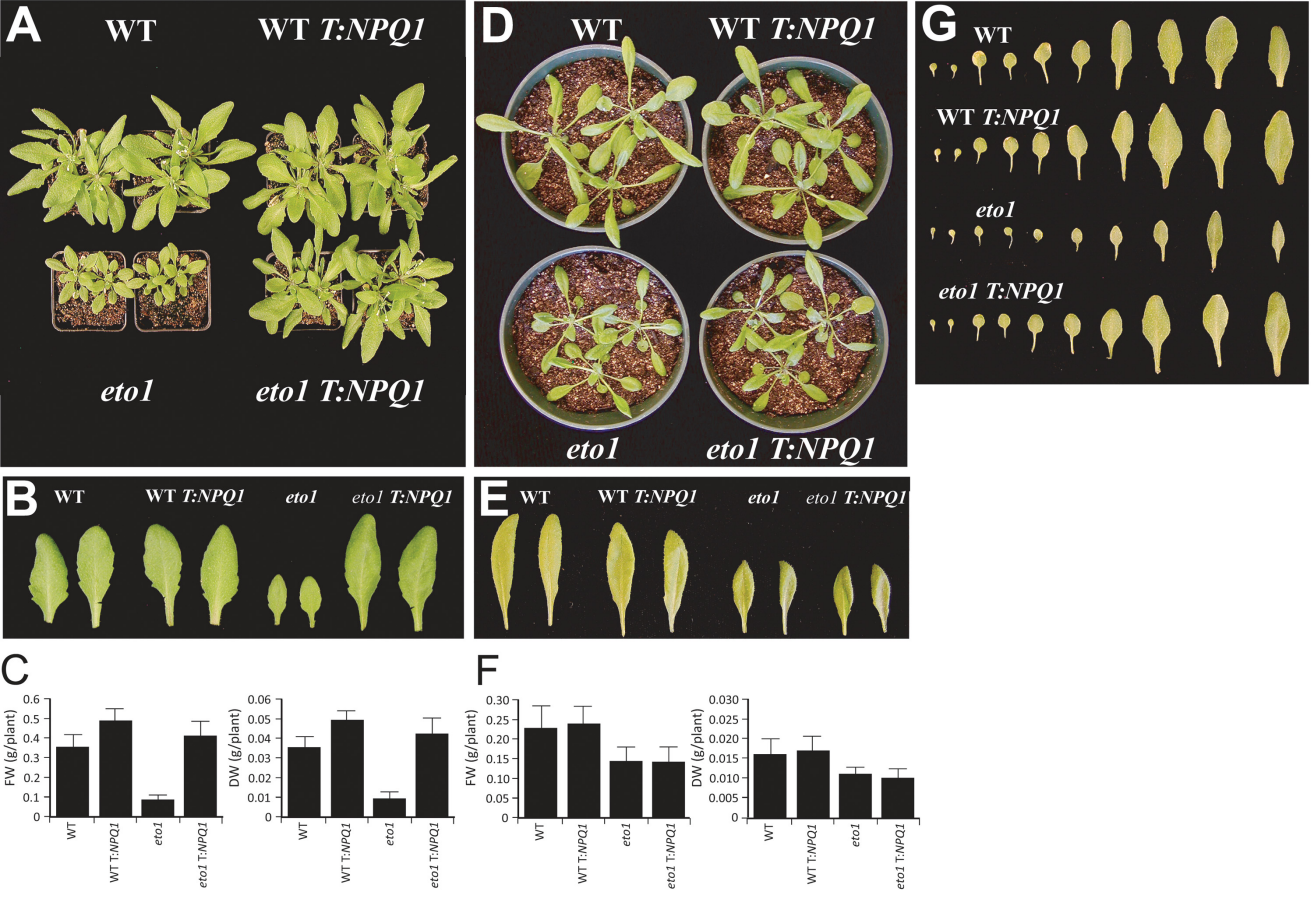
Restoring VDE expression reverses the small growth phenotype of *eto1-1* plants. (A) *eto1-1* and WT plants without or with the 35S::*NPQ1* transgene were grown at 1200 PFD for 3.5 weeks. (B) Comparison of adult rosette leaves of *eto1-1* and WT plants with or without the 35S::*NPQ1* transgene grown at 1200 PFD. (C) Fresh and dry weight of *eto1-1* and WT plants without or with the 35S::*NPQ1* transgene grown at 1200 PFD. (D) *eto1-1* and WT plants without or with the 35S::*NPQ1* transgene were grown under 50 PFD for 3.5 weeks. (E) Comparison of adult rosette leaves of *eto1-1* and WT plants with or without the 35S::*NPQ1* transgene grown under 50 PFD. (F) Fresh and dry weight of *eto1-1* and WT plants without or with the 35S::*NPQ1* transgene grown at 50 PFD. (G) Every leaf from *eto1-1* and WT plants without or with the 35S::*NPQ1* transgene grown for 3.5 weeks at 1200 PFD.

Exposure to high light induces ROS synthesis and can result in growth retardation [125]. Therefore, the higher rate of O_2_^.-^ production and increased photoinhibition experienced by in *eto1-1* and *ctr1-3* may negatively affect their growth [49, 107]. *ctr1-3* is considerably smaller than *eto1-1*, correlating with its higher level of ethylene signaling, and their small stature has been attributed to a smaller cell size [14, 80, 81]. If the greater photosensitivity of *eto1-1* and *ctr1-3* contributes to their small growth phenotype when grown under high light, restoring VDE activity should not only reduce ROS generation and photoinhibition but may also reverse the small growth phenotype of *eto1-1* and *ctr1-3* to some extent. To test this, we grew WT, *eto1-1*, and *ctr1-3* plants with or without the 35S::*NPQ1* transgene under high light and compared their growth characteristics. *eto1-1* T:*NPQ1* plants were substantially larger than *eto1-1* plants when grown under high light (Fig 9A) which resulted from an increase in leaf size (Fig 9B) and cell size (S4A Fig). Increasing VDE expression in WT plants increased plant size to a much smaller extent than it did in *eto1-1* plants (Fig 9A and 9B, S4A Fig). Quantitative measurements revealed that the fresh and dry weights of *eto1-1* T:*NPQ1* were 4.6-fold and 4.2-fold greater, respectively, than *eto1-1* plants (Fig 9C). Consequently, *eto1-1* T:*NPQ1* plants were similar in stature and biomass to WT plants. In contrast, increasing VDE expression in WT plants increased their fresh and dry weights by just 30% and 42%, respectively (Fig 9C). Comparison of all leaves from *eto1-1* T:*NPQ1* and *eto1-1* plants revealed that increasing VDE expression in *eto1-1* did not alter cotyledon size but it did increase the size of all true leaves (Fig 9G). Increasing VDE expression in *ctr1-3* also increased plant stature when grown at 250 PFD but to a smaller extent than observed in *eto1-1* plants (S5 Fig). *ctr1-3* T:*NPQ1* plants had larger leaves and retained a greater number of leaves at flowering than did *ctr1-3* plants (S5 Fig).

If the restoration of VDE activity reversed the small growth phenotype of *eto1-1* because of a reduction in photodamage during growth under high light, restoring VDE activity in *eto1-1* would be predicted to have a substantially smaller effect on growth under low light which limits ROS production and photodamage (Table 3). To test this hypothesis, we grew the same lines under low light (i.e., 50 PFD). *eto1-1* T:*NPQ1* plants were similar in size to *eto1-1* plants (Fig 9D) as was the size of leaves under these growth conditions (Fig 9E). The fresh and dry weights of *eto1-1* T:*NPQ1* were not significantly different from *eto1-1* under these growth conditions (Fig 9F). The presence of the transgene also had little effect on the growth of WT plants (Fig 9D and 9E) or the fresh or dry weights of WT plants (Fig 9F). The fresh weight of *eto1-1* plants, however, was 66% of WT when grown under low light versus 25% of WT when grown under high light (compare Fig 9F to 9C) indicating that high light has a disproportionately negative effect on *eto1-1* growth. Similarly under low light, *ctr1-3* T:*NPQ1* fresh weight (0.010 g) was not significantly different from that of *ctr1-3* (0.010 g). Thus, the reversal of the small stature of *eto1-1* and *ctr1-3* following restoration of VDE activity was observed specifically under high light conditions. These results suggest that increases in ethylene responses cause an increase in ROS production during exposure to high light that contributes to a reduction in stature. The data also suggest that restoring VDE activity lowers ROS production and reduces their deleterious effect on plant stature.

### Increasing VDE expression does not alter ethylene production or ethylene responses

The effect that increasing VDE expression had on reversing the small cell size and plant stature of *eto1-1* raised the possibility that the increase in VDE expression may reduce ethylene production. To examine this, we measured the production of ethylene in *eto1-1* and *eto1-1* T:*NPQ1* plants. Ethylene production was nearly 4-fold higher in *eto1-1* plants than in WT but ethylene production in *eto1-1* T:*NPQ1* plants was not significantly different from that in *eto1-1* (S6A Fig). Increasing VDE expression in WT plants also did not affect ethylene evolution (S6A Fig).

We next investigated the effect of VDE on ethylene responsiveness by examining the triple response of seedlings to ethylene when grown on medium with or without ACC, the precursor to ethylene. The triple response of Arabidopsis is an ethylene-mediated response of dark-grown seedlings characterized by the radial expansion of the hypocotyl, inhibition of root and hypocotyl elongation, and the presence of an exaggerated apical hook [126]. In the absence of ACC, seedling growth is influenced by the endogenous production of ethylene only. The hypocotyls and roots of *eto1-1* were substantially shorter than WT and exhibited a more pronounced apical hook than WT (Table 7 and S6B Fig), consistent with its higher production of ethylene (S6A Fig). Growth of *eto1-1* T:*NPQ1* seedlings was not significantly different from *eto1-1* seedlings and the pronounced apical hook remained (Table 7 and S6B Fig). Increasing VDE expression in WT plants also did not alter the triple response of WT seedlings (Table 7 and S6B Fig). However, because VDE is light-activated, growth of light-grown seedlings was also examined. Although light-grown seedlings differ in their response to ethylene from those grown in the dark, the light-grown seedling response is characterized by a reduced cotyledon size, a delay in true leaf emergence, and an inhibition of root growth [127]. To examine the effect that VDE has on ethylene responsiveness in light-grown seedlings where VDE would be active, we germinated the same lines on medium with or without 20 μM ACC and grown in low light (i.e., 100 PFD) to avoid photoinhibition. In the absence of ACC, *eto1-1* T:*NPQ1* seedlings exhibited a similar reduction in cotyledon size and root length as *eto1-1* (S6C and S6D Fig). The size of cotyledons and roots of WT T:*NPQ1* seedlings were similar to those of WT T:*NPQ1* (S6C and S6D Fig). In the presence of ACC, all lines exhibited small cotyledons and short roots at the cotyledon stage (S6C and S6D Fig) and following emergence of the first pair of true leaves (S6E Fig). These results indicate that the expression of VDE does not affect ethylene production or ethylene responses.

**Table 7:** Increasing VDE expression does not affect ethylene production or responsiveness.

	Root length^a^ (mm)				Ethylene production^b^ (nl/g/hr)			
	No ACC	t-test	20 μM ACC	t-test	250 PFD	t-test	1900 PFD	t-test
WT	8.67 ± 0.62		2.58 ± 0.53		10.1 ± 0.3		11.3 ± 0.6	
WT *T*:*NPQ1*	8.50 ± 0.67	P = 0.602	2.73 ± 0.39	P = 0.517	10.6 ± 0.2	P = 0.148	12.0 ± 0.7	P = 0.345
*eto1*	6.00 ± 0.61	P<0.001	2.27 ± 0.39	P = 0.196	38.1 ± 0.4	P<0.001	22.9 ± 1.2	P<0.001
*eto1 T*:*NPQ1*	6.00 ± 0.91	P<0.001	2.32 ± 0.32	P = 0.246	37.5 ± 1.3	P<0.001	19.2 ± 0.9	P<0.001

^a^Measurements taken from 4 day old seedlings. The average of 20–30 seedlings and standard deviation for each are reported.

^b^Measurements taken from four replicates of adult leaves. The average and standard deviation for each are reported.

## Discussion

In this study, we show that *eto1-1*, in which ethylene production is increased moderately, and *ctr1-3*, in which ethylene responses are constitutive exhibit an impaired qE that is due, in part, to a reduction in VDE activity. This conclusion is supported by the observation that *eto1-1* and *ctr1-3* exhibit significantly lower rates of light-induced de-epoxidation that could be corrected by restoring VDE activity. The reduced de-epoxidation activity in *eto1-1* was explained by a reduction in VDE activation while the larger reduction in de-epoxidation in *ctr1-3* was explained by a 2-fold reduction in the levels of *NPQ1* mRNA, VDE protein, VDE enzyme activity, as well as a reduction in the activation of VDE activity.

*NPQ1* promoter activity was repressed following an increase in ethylene responses, imposed either pharmacologically or genetically (Fig 4). The repression of *NPQ1* promoter activity correlated with the level of ethylene responses in that its activity was repressed to greater extent in *ctr1-3* than in *eto1-1*. This conclusion is consistent with the level of ethylene required to repress *NPQ1* expression in WT plants in that *NPQ1* promoter activity was repressed approximately 2-fold following a 10.6-fold increase in ethylene production by exogenous application of ACC compared to the more modest reduction in promoter activity observed in *eto1-1* which exhibits less than a 4-fold increase in ethylene production under moderate light. Although the reduction in *NPQ1* promoter activity in *ctr1-3* partially accounted for the reduction in *NPQ1* transcript and VDE protein levels, the small reduction in *NPQ1* promoter activity in *eto1-1* was not reflected in reduced *NPQ1* transcript or VDE protein levels (Fig 3). Nevertheless, the substantially lower level of de-epoxidation activity present in *eto1-1* indicated a reduction in VDE enzyme activity, which requires an acidified thylakoid lumen generated by light-induced proton pumping from the stroma for its activation.

Direct measurements revealed that *eto1-1* and *ctr1-3* are unable to establish a full transthylakoid membrane pH gradient during exposure to saturating light and that restoring VDE activity substantially reversed this defect (Table 4). The lower transthylakoid membrane ΔpH in *eto1-1* and *ctr1-3* was not a result of a reduction in electron flow as their ETR was actually higher than WT, suggesting a defect in establishing or maintaining a transthylakoid membrane pH gradient. Under nonsaturating light, however, *eto1-1* was able to establish a transthylakoid membrane ΔpH equivalent to WT while the transthylakoid membrane ΔpH in *ctr1-3* was closer to the WT value under nonsaturating light than it was under saturating light. This suggests that the defect in establishing a full transthylakoid membrane pH gradient in *eto1-1* occurs specifically during exposure to elevated light levels and is exacerbated in *ctr1-3* under these same conditions.

These observations also indicate that despite the higher rate of electron flow through PSII in these mutants, an increase in ethylene responses results in a lower transthylakoid membrane ΔpH, a reduction in VDE activation, and a greater proportion of electrons being used for ROS generation, particularly under saturating light. This possibility was supported by the substantially higher rate of O_2_^.-^ generation in *eto1-1* and *ctr1-3* and the accompanying higher level of photoinhibition experienced by these mutants under saturating light but not under low light relative to WT (Table 3). *npq1*, which lacks VDE expression, as well as *npq4*, which lacks PsbS expression, also exhibit higher rates of O_2_^.-^ production when exposed to saturating light (Table 3), supporting the conclusion that the increase in O_2_^.-^ production in the ethylene mutants results, in part, from a reduction in xanthophyll cycle functioning. That the level of O_2_^.-^ production in *eto1-1* and *ctr1-3* is higher than the levels in *npq1* or *npq4*, however, suggests that the reduced functioning of the xanthophyll cycle in *eto1-1* and *ctr1-3* only partially accounts for the increase in ROS production during exposure to saturating light. Nevertheless, the partial reversal of the aberrantly high level of O_2_^.-^ production in *eto1-1* (Table 3) and the generation of a normal transthylakoid membrane pH gradient (Table 4) following restoration of VDE activity specifically during exposure to high light supports the notion that the ethylene-mediated reduction in VDE activity does contribute to these defects.

The generation of a transthylakoid membrane ΔpH is necessary for the activation of VDE enzyme activity and the generation of qE [51, 52, 105, 128]. The light-mediated acidification of the thylakoid lumen causes protonation of specific amino acid residues in VDE that alters its conformation and causes the enzyme to dock with the thylakoid membrane where it comes in contact with V and de-epoxidates it to A and Z [51, 52]. A low thylakoid lumen pH may also promote a conformational change in the membrane [129]. A more basic thylakoid lumen and more acidic stroma would have the potential to affect several chloroplast functions critical to NPQ, including lower activation of VDE, reduced de-epoxidation, and impaired qE.

As protonation of PsbS under conditions of excess light is necessary for its function [130], its activity, like VDE, requires an acidified thylakoid lumen. Defects in establishing or maintaining a protein gradient across the thylakoid membrane would be expected to reduce PsbS function, as well as that of VDE, thus leading to an impaired qE. Although the more acidic stroma of *eto1-1* prevents full activation of VDE activity as measured by its decreased rate of de-epoxidation, it is unknown whether it is sufficient to affect PsbS activity. PsbS expression, however, does determine qE capacity [107] and a reduction in PsbS levels are accompanied by changes in the structural organization of PSII-LHCII arrays in the thylakoid membrane [131, 132, 133]. Therefore, the observed reduction in PsbS expression and perhaps activation may contribute to the lower qE observed in *eto1-1* and *ctr1-3*.

Acidification of the thylakoid lumen during light exposure also results in protonation of certain light-harvesting antenna complexes (LHCs) as well as causing conformational changes in the LHCs that facilitate qE [134, 135, 136]. Zeaxanthin is important in promoting the dissociation of LHCII from PSII and its aggregation [136]. Zeaxanthin has also been proposed to bind LHCII proteins to either quench excited chlorophyll directly or to function as an allosteric modulator of the ΔpH-sensitive qE inherent in LHCII proteins [56, 66, 137]. The link between pH-induced LHC II aggregation and qE can be observed *in vitro* with isolated LHCs [134].

A more acidic stroma would also be expected to reduce the level of soluble CO_2_, which in turn would be expected to reduce the rate of CO_2_ assimilation. The observation that the observed lower rate of CO_2_ assimilation in *eto1-1* can be corrected by increasing the internal CO_2_ concentration (Fig 5A) supports the notion that a more acidic stroma limits CO_2_ solubility. Thus, a more acidic stroma in *eto1-1* (and *ctr1-3*) could affect multiple processes that contribute to the observed defects in VDE activation, xanthophyll de-epoxidation, qE, CO_2_ assimilation, as well as the increase in ROS generation. The observation that restoring VDE activity to *eto1-1* restores near WT levels of qE is again consistent with the conclusion that the ethylene-mediated reduction in VDE activation contributes to the reduction in qE.

Although the defects in the expression and activation of VDE in *eto1-1* and *ctr1-3* were consistent with the observed reduced initial induction of NPQ, the level of NPQ eventually overtook the steady-state WT level following prolonged exposure to light (S1 Fig). The initial induction of NPQ typically represents qE whereas qI largely contributes to the additional accumulation in NPQ following its initial rapid rise. The increase in NPQ in *eto1-1* and *ctr1-3* that eventually exceeds the WT steady-state level, therefore, can be understood as an increase in photoinhibitory processes during prolonged exposure to higher light levels. An increase in qI was consistent with a lower relative F_v_/F_m_ (Fig 8B and 8C), a lower NPQ_f_ and higher NPQ_s_ (Figs 1G and 8A), and a slower rate of recovery following high light stress (Fig 8B and 8C). That an increase in ethylene responses was responsible for the accumulation in qI was supported by the observation that inhibiting ethylene perception in *eto1-1* largely prevented qI accumulation (S1D Fig). Moreover, preventing repair of damaged PSII reaction centers through the inhibition of new protein synthesis revealed that repair plays a disproportionately larger role in *eto1-1* and *ctr1-3* (Fig 8D).

A reduction in photoprotection caused by an impaired xanthophyll cycle would be expected to be exacerbated by a reduction in CO_2_ solubility in the stroma as both would contribute to an increase in ROS and photoinhibition. Accordingly, the increase in qI in *eto1-1* and *ctr1-3* could be prevented by either increasing the concentration of CO_2_ (Figs 5B and 7B) or by restoring VDE activity (Figs 7A and 8). The notion that an increase in ethylene responses reduces the level of soluble CO_2_ in the stroma is also supported by photosynthetic measurements in which the reduced rate of CO_2_ assimilation in *eto1-1* could be corrected by increasing the level of soluble CO_2_ (Fig 5A). A similar stomatal conductance in *eto1-1* and WT excluded the possibility of reduced gas diffusion. The observation that *eto1-1* required a higher C_i_ in order to achieve a ½V_max_ rate of CO_2_ assimilation equivalent to WT was consistent with a more acidic stroma as suggested by the transthylakoid pH gradient measurements in this mutant.

These observations indicate that the level of soluble CO_2_ present in the stroma of *eto1-1* and *ctr1-3* is lower than in WT. As CO_2_ serves as the final electron sink in photosynthesis, a reduction in the level of soluble CO_2_ may result in the over reduction of the photosystems and an accumulation of photoinhibition during prolonged exposure to light, thus contributing to the increase in NPQ in *eto1-1* and *ctr1-3* that eventually overtakes the steady-state WT level. Such a scenario can also occur during drought conditions where water stress-induced stomatal closure limits the diffusion of CO_2_ into chloroplasts. This leads to decreased CO_2_ assimilation, increased photorespiration, and elevated H_2_O_2_ generation. Increased production of singlet oxygen and O_2_^.-^, the latter of which can be generated by the water-water-cycle associated with PSI or by oxygen reacting with reduced quinones on the acceptor-side of PSII [44, 138, 139, 140], also occurs under conditions of limiting CO_2_ [43, 141]. The elevated level of O_2_^.-^ in *eto1-1* and *ctr1-3* specifically during exposure to high light correlates with the accumulation of photoinhibition in these mutants whereas the restoration of VDE activity reduced the level in photoinhibition (Fig 8) as it did the aberrantly high level of O_2_^.-^ (Table 3).

The reduced cell size and plant stature of *eto1-1* and *ctr1-3* are likely a consequence of a combination of regulation by ethylene directly as well as indirectly through a reduction in qE, an increase in O_2_^.-^ generation and qI, and a reduction in net photosynthetic gain. The extent to which photoinhibitory processes and ROS are responsible for the reduction in cell size and plant stature should be observed only under those conditions resulting in their elevated production, e.g., following exposure to excess light. The observation that restoring VDE expression in *eto1-1* reversed its small cell size and plant stature specifically under moderate to high light, but not low light when ROS levels are low (Fig 9), supports the conclusion that increased ethylene signaling results in photodamage from elevated ROS to which an impaired xanthophyll cycle likely contributes. Although *eto1-1* plants are also smaller than WT when grown under non-photoinhibitory conditions, they are considerably closer in size to WT during growth under low light (66% of WT fresh weight) compared to growth under high light (25% of WT fresh weight) (Fig 9), demonstrating that the degree to which increased ethylene signaling reduces plant stature is exacerbated by growth under high light. These observations suggest that ethylene regulates cell size in part through impairment of the xanthophyll cycle under high light conditions in addition to its role in regulating cell size under lower light conditions.

The higher level of ethylene signaling in *ctr1-3* results in an even smaller cell size and reduced plant stature than in *eto1-1*. VDE and PsbS expression are repressed in *ctr1-3*, resulting in a reduced rate of de-epoxidation and qE. Restoring VDE expression increased cell size when the mutant was grown under moderate light but this was offset by a decrease in the number of cells per leaf such that no change in plant stature was observed. As *ctr1-3* could not be grown to adulthood under high light, it wasn’t possible to examine the effect of increasing VDE expression on *ctr1-3* growth under the same conditions used for *eto1-1*. It is possible, however, that the direct regulation of cell size by the high level of ethylene signaling in *ctr1-3* makes a greater contribution to its small stature than does its impaired xanthophyll cycle.

The reduction in VDE activity in *eto1-1* and *ctr1-3* is not solely responsible for the small growth phenotype of these mutants as *npq1* (or *npq4*) is not substantially smaller than WT [124]. However, the fact that restoring VDE activity in *eto1-1* can fully reverse its small stature and partially reverse the small stature of *ctr1-3* suggests that a reduction in VDE activity in the context of increased ethylene responses does contribute to a reduction in growth specifically under conditions of high light. The effect of restoring VDE activity on growth of in *eto1-1* and *ctr1-3* was specific to growth under high light as demonstrated by its failure to reverse the small growth phenotype of these mutants under low light. This finding also supports the conclusion that ROS contributes to the small growth phenotype of *eto1-1* and *ctr1-3* under high light as ROS production increases substantially in both mutants to a greater extent than in WT but is reversed by restoring VDE activity.

The negative effect that ROS has on growth was also observed in Arabidopsis silenced for the zinc finger transcription factor *ZAT10*, which was characterized by elevated levels of H_2_O_2_ and O_2_^.-^ and reduced photochemistry when exposed to high light, resulting in substantial growth retardation [142]. Similarly, loss of ETHYLENE RESPONSE FACTOR 6 expression resulted in elevated levels of H_2_O_2_, photosensitivity, and reduced growth [143, 144].

The importance of VDE activity in photoprotection is most clearly demonstrated with the *npq1* mutant which lacks VDE expression altogether. *npq1* exhibits substantially greater photoinhibition following transfer to high light than WT as measured by a lower F_v_/F_m_, reduced quantum yield of electron transport (φPSII), lower rate of CO_2_ assimilation (despite a similar rate of transpiration), and some reduction in growth [49]. These phenotypes were even more pronounced when combined with the *npq4* mutation [49, 107]. *npq1* plants also exhibited photooxidative damage, including bleaching and areas of necrosis following sudden exposure to high light [145]. *npq1* (and *npq4*) plants exhibited reduced fitness when grown under field conditions involving exposure to full sun or in rapidly fluctuating moderate light [146], demonstrating that changes to VDE (or PsbS) expression affect growth under variable light environments. When grown in high light, however, *npq1* exhibited growth similar to WT suggesting acclimation that may have been supported by the higher levels of α-tocopherol in young leaves of this mutant that protected against ROS-mediated damage [73, 124, 147]. *npq4* also exhibits an increase in the level of α-tocopherol as well as an increase in ascorbic acid content [124]. The significant increase in α-tocopherol content in *npq1* (and to a lesser extent *npq4*) was confirmed in our study (Table 6). In contrast, *eto1-1* exhibited no such increase in α-tocopherol or ascorbic acid so that the reduction in its VDE activity and PsbS expression occurred in the absence of any additional compensating antioxidants. Moreover, zeaxanthin and α-tocopherol exhibit synergistic protection against photodamage [15, 148, 149, 150] so a decrease in one may be amplified in the absence of an increase in the other. *ctr1-3* exhibited an increase in α-tocopherol content (but not in ascorbic acid content) similar to *npq1*, raising the possibility that the increase in α-tocopherol may have partially compensated for the greater reduction in VDE activity and PsbS expression relative to *eto1-1* despite its much higher level of ethylene signaling.

Although the reduction in VDE activity in *eto1-1* and *ctr1-3* is responsible for their reduced de-epoxidation and likely contributes to their increased ROS levels, it is not solely responsible for their increased oxidative load as restoration of VDE activity in *eto1-1* and *ctr1-3* only partially reversed the increase in ROS. Although the level of ROS is elevated in *npq1*, it does not reach the levels observed in *eto1-1* and *ctr1-3* (Table 3). Additionally, restoration of VDE activity in *ctr1-3* only partially reversed its small cell phenotype. Such observations indicate that the reduction in VDE activity in the context of increased ethylene signaling does increase photosensitivity, ROS generation, and growth impairment as demonstrated by the reversal of these effects following the restoration of VDE activity. These observations also show the effects of restoring VDE activity was specific to *eto1-1* and *ctr1-3* as increasing VDE expression in WT plants did not have the same effect. However, because restoring VDE expression failed to fully reverse ROS generation and photosensitivity in *eto1-1* and *ctr1-3*, ethylene likely affects other factors that also contribute to the oxidative load. We can conclude, however, that ethylene does negatively regulate the expression and activity of VDE and this repression impairs the function of the xanthophyll cycle while contributing to the increased photosensitivity and reduced plant growth specifically under high light conditions. An important area for future work will be to determine whether ethylene regulates NPQ and VDE expression through similar mechanisms in other species to establish whether the observations made in Arabidopsis are common throughout plant species.

## Supporting Information

S1 Fig Elevated Ethylene Signaling Results in Aberrant Induction of NPQ. The kinetics of NPQ induction in 3 week-old *eto1-1*, *ctr1-3*, and WT plants exposed to (A) 400 μmol photons m^-2^ s^-1^ for 25 min, (B) 400 μmol photons m^-2^ s^-1^ for 6 min, or (C) 100 μmol photons m^-2^ s^-1^ for 8 min was measured following the transfer of dark-adapted plants to light. WT (filled diamonds); *eto1-1* (filled circles); *ctr1-3* (open diamonds). (D) The kinetics of NPQ induction in 3 week-old *eto1-1* and WT plants treated with 1-MCP or air for 20 hr was measured following the exposure of dark-adapted plants to 400 μmol photons m^-2^ s^-1^. NPQ *eto1-1* was determined by (Fm-Fm’)/Fm’. WT (filled diamonds); WT + 1-MCP (open diamonds); (filled circles); *eto1-1* + 1-MCP (open circles).
(EPS)

S2 Fig Restoring VDE Expression Corrects the Aberrant NPQ Induction in *eto1-1* and *ctr1-3* Plants. (A) The kinetics of NPQ induction in 3 week-old WT, *eto1-1*, and *ctr1-3* plants with or without the 35S::NPQ1 transgene following their exposure to 1000 μmol photons m^-2^ s^-1^ for 4.5 min and its relaxation following transfer to darkness for an additional 100 sec. (B) The early induction kinetics of NPQ from (A) to show the initial rate of NPQ induction in the same lines following their exposure to 1000 μmol photons m^-2^ s^-1^. Induction of NPQ in the same lines when 3 weeks old following their exposure to (C) 100 or (D) 400 μmol photons m^-2^ s^-1^. WT (open diamonds); WT T:NPQ1 (filled diamonds); *eto1-1* (open squares); *eto1-1* T:NPQ1 (filled squares); *ctr1-3* (open circles); *ctr1-3* T:NPQ1 (filled circles).
(EPS)

S3 Fig Restoring VDE Expression Partially Corrects the Aberrant Electron Transport Rate in *eto1-1* and *ctr1-3* Plants. (A) The electron transport rate (ETR) was measured in WT, *eto1-1*, and *ctr1-3* plants with or without the 35S::NPQ1 transgene as a function of photon flux density (PFD). (B) The ETR was measured in WT, *eto1-1*, *ctr1-3*, *npq1*, and *npq4* plants.
(EPS)

S4 Fig Restoring VDE Expression Reverses the Small Cell Size of *eto1-1* Plants Without Affecting the Stomatal Index. SEM analysis of epidermal cells from the adaxial surface of adult rosette leaves of *eto1-1* and WT plants with or without the 35S::NPQ1 transgene grown for 3 weeks in sunlight.
(EPS)

S5 Fig Restoring VDE Expression Increases Biomass of *ctr1-3*. (A) *ctr1-3* plants with or without the 35S::NPQ1 transgene were grown under high light (i.e., 1000 μmol photons m^-2^ s^-1^) until flowering. (B) Adult leaves of *ctr1-3* plants with or without the 35S::NPQ1 transgene grown under 1000 PFD. (C) Every leaf (left to right: oldest to youngest) from *ctr1-3* plants with or without the 35S::NPQ1 transgene grown under 1000 PFD until flowering.
(EPS)

S6 Fig VDE Does Not Affect Ethylene Production or Ethylene Responses. (A) Ethylene evolution from 3 week-old *eto1-1* and WT plants with or without the 35S::NPQ1 transgene. (B) Triple response assay of *eto1-1* and WT lines with or without the 35S::NPQ1 transgene germinated on medium with or without 20 μM ACC and grown for 4 days in the dark. (C) Light-grown ethylene response assay of *eto1-1* and WT lines with or without the 35S::NPQ1 transgene germinated on medium with or without 20 μM ACC and grown in low light (i.e., 100 μmol photons m^-2^ s^-1^). (D) Representative individual seedlings from the light-grown ethylene response assay in which seedlings were germinated with or without ACC to show the reduced cotyledon size of the *eto1-1* relative to WT and that the presence of the 35S::NPQ1 transgene in *eto1-1* or WT does not affect cotyledon size. (E) Representative individual seedlings from the same light-grown ethylene response assay in (D) following an additional 5 days of growth to show that the presence of the 35S::NPQ1 transgene in *eto1-1* or WT does not affect plant stature under low light or the epinastic response in the presence of ACC.
(EPS)

S1 Table Ascorbate Pool Size and Redox State in WT, *eto1*, and *ctr1* Overexpressing VDE. (DOC)
