# Supplementary material for: Ethylene Regulates Energy-Dependent Non-Photochemical Quenching in Arabidopsis through Repression of the Xanthophyll Cycle
Source: PLoS One. 2015 Dec 2;10(12):e0144209. doi: 10.1371/journal.pone.0144209 (PMC4667945; doi:10.1371/journal.pone.0144209)
Supplement: S1 Table — (DOC) [file pone.0144209.s007.doc]

**Supplemental Table 1.** Restoring VDE expression to *eto1-1* and *ctr1-3* does not alter their ascorbate pool size or redox state

|  | Light-adapted^a^ | | | 2 hr sunlight^b^ | | |
| --- | --- | --- | --- | --- | --- | --- |
|  | Asc  (μmol/g FW) | Asc + DHA  (μmol/g FW) | Asc redox state  [Asc/(Asc + DHA)] | Asc  (μmol/g FW) | Asc + DHA  (μmol/g FW) | Asc redox state  [Asc/(Asc + DHA)] |
| WT | 4.10 ± 0.14 | 5.14 ± 0.08 | 0.797 | 3.85 ± 0.08 | 5.20 ± 0.08 | 0.741 |
| WT *T:NPQ1* | 4.28 ± 0.27 | 5.48 ± 0.11 | 0.782 | 3.92 ± 0.07 | 5.35 ± 0.17 | 0.733 |
| *eto1* | 4.12 ± 0.23 | 5.30 ± 0.10 | 0.777 | 3.89 ± 0.07 | 5.28 ± 0.28 | 0.736 |
| *eto1* *T:NPQ1* | 4.25 ± 0.08 | 5.35 ± 0.03 | 0.794 | 3.99 ± 0.07 | 5.37 ± 0.20 | 0.742 |
| *ctr1* | 4.18 ± 0.23 | 5.26 ± 0.14 | 0.794 | 3.84 ± 0.17 | 5.32 ± 0.26 | 0.722 |
| *ctr1 T:NPQ1* | 4.04 ± 0.26 | 5.14 ± 0.15 | 0.786 | 3.81 ± 0.12 | 5.26 ± 0.15 | 0.726 |

^a^Determined from four replicates of adult leaves grown at 250 PFD for three weeks that were light-adapted at 250 PFD for 4 hr.

^b^Determined from four replicates of adult leaves grown at 250 PFD for three weeks that were exposed to 2 hr sunlight (1900 PFD).
